# Sulcal morphology of ventral temporal cortex is shared between humans and other hominoids

**DOI:** 10.1038/s41598-020-73213-x

**Published:** 2020-10-13

**Authors:** Jacob A. Miller, Willa I. Voorhies, Xiang Li, Ishana Raghuram, Nicola Palomero-Gallagher, Karl Zilles, Chet C. Sherwood, William D. Hopkins, Kevin S. Weiner

**Affiliations:** 1 Helen Wills Neuroscience Institute, 210 Barker Hall, University of California, Berkeley, Berkeley, CA 94720 USA; 2 Department of Psychology, University of California, Berkeley, Berkeley, CA 94720 USA; 3 School of Clinical Sciences, University of Edinburgh, Edinburgh, UK; 4 Research Centre Jülich, Institute of Neuroscience and Medicine INM-1, Jülich, Germany; 5 Department of Psychiatry, Psychotherapy and Psychosomatics, Medical Faculty, RWTH Aachen University, Aachen, Germany; 6 C. & O. Vogt Institute for Brain Research, Heinrich-Heine-University, 40225 Düsseldorf, Germany; 7 JARA-Translational Brain Medicine, Aachen, Germany; 8 Department of Anthropology and Center for the Advanced Study of Human Paleobiology, The George Washington University, 800 22nd Street NW, Suite 6000, Washington, DC 20052 USA; 9 Department of Comparative Medicine, The University of Texas MD Anderson Cancer Center, Bastrop, TX 78602 USA

**Keywords:** Perception, Neuroscience

## Abstract

Hominoid-specific brain structures are of particular importance in understanding the evolution of human brain structure and function, as they are absent in mammals that are widely studied in the extended neuroscience field. Recent research indicates that the human fusiform gyrus (FG), which is a hominoid-specific structure critical for complex object recognition, contains a tertiary, longitudinal sulcus (mid-fusiform sulcus, MFS) that bisects the FG into lateral and medial parallel gyri. The MFS is a functional and architectonic landmark in the human brain. Here, we tested if the MFS is specific to the human FG or if the MFS is also identifiable in other hominoids. Using magnetic resonance imaging and cortical surface reconstructions in 30 chimpanzees and 30 humans, we show that the MFS is also present in chimpanzees. The MFS is relatively deeper and cortically thinner in chimpanzees compared to humans. Additional histological analyses reveal that the MFS is not only present in humans and chimpanzees, but also in bonobos, gorillas, orangutans, and gibbons. Taken together, these results reveal that the MFS is a sulcal landmark that is shared between humans and other hominoids. These results require a reconsideration of the sulcal patterning in ventral temporal cortex across hominoids, as well as revise the compensation theory of cortical folding.

## Introduction

The evolution of human brain structure and function is of major interest in comparative biology and systems neuroscience. To understand the evolutionary emergence of neuroanatomical structures observed in humans, brains from across primate phylogeny must be sampled, with a particular emphasis on hominoids (i.e., apes, which includes gibbons, siamangs, orangutans, gorillas, bonobos, chimpanzees, and humans). The hominoid branch originated approximately 20 million years ago, and the lineage leading to humans diverged 6–8 million years ago^1,2^. Thus, to map the evolution of human brain structure, comparisons to other hominoids, such as chimpanzees, are essential. Hominoid-specific brain structures are particularly intriguing because they are absent in mammals that are widely studied in the extended neuroscience field, such as mice, marmosets, and macaques. Several hominoid-specific brain structures have been identified in the temporal lobe, including the planum temporale and fusiform gyrus (FG)^3–5^.

The fusiform gyrus (FG) is critical for different aspects of what is often referred to as “high-level” visual processing, which includes object recognition^6–11^, face perception^12–21^, and reading^22–25^ in humans. A shallow, longitudinal sulcus known as the mid-fusiform sulcus (MFS;^26^ for review) bisects the human FG into lateral and medial partitions. Recent studies^27–30^ have revealed that the MFS is a microanatomical and functional landmark in human association cortex that is causally implicated in visual perception^16,17^. Nevertheless, despite the utility of the MFS for understanding the relationship among structure, function, and perception in humans^26^, it is presently unknown if the MFS is unique only to humans or if it is also present in other hominoids, which has important implications for understanding the evolution of ventral temporal cortex (VTC).

We sought to examine the sulcal patterning of VTC in structural magnetic resonance imaging (MRI) scans and cortical surface reconstructions of chimpanzee brains and other hominoids compared to humans. We asked four main questions. First, do chimpanzees have a mid-fusiform sulcus (MFS)? Second, if the MFS is present in chimpanzees, are morphological features such as mean depth, max depth (i.e. sulcal pit^31–33^), cortical thickness, and length of the MFS similar or different between chimpanzees and humans? Third, is VTC sulcal morphology similar enough between humans and chimpanzees that cortex-based alignment (CBA) can predict the location of individual sulci within VTC across species? Specifically, can VTC sulci defined on a human cortical surface template predict the location of VTC sulci in individual chimpanzees (and vice versa)? Fourth, is the MFS identifiable in histological sections and cortical surface reconstructions in other apes?

Our approach revealed that the sulcal morphology of VTC is shared between humans and other hominoids. The MFS is identifiable in chimpanzees and like the human MFS^26,29^, the most stable and variable morphological features of the chimpanzee MFS are (a) its shallowness relative to surrounding VTC sulci and (b) its length, respectively. Interestingly, the MFS is relatively deeper and relatively cortically thinner in chimpanzees compared to humans and its location can be predicted equally well in chimpanzees using either a human or chimpanzee template. Finally, histological analyses reveal that the MFS is also identifiable in bonobos, gorillas, orangutans, and gibbons. The shallowness of the MFS relative to neighboring sulci is a stable morphological feature across species. Taken together, these results reveal that the MFS is a sulcal landmark that is shared across hominoids. We discuss these results in the context of the evolution of both cortical folding and functionally-specialized maps and regions within high-level visual cortex.

## Results

### The MFS is identifiable in chimpanzees

**Figure 1:**
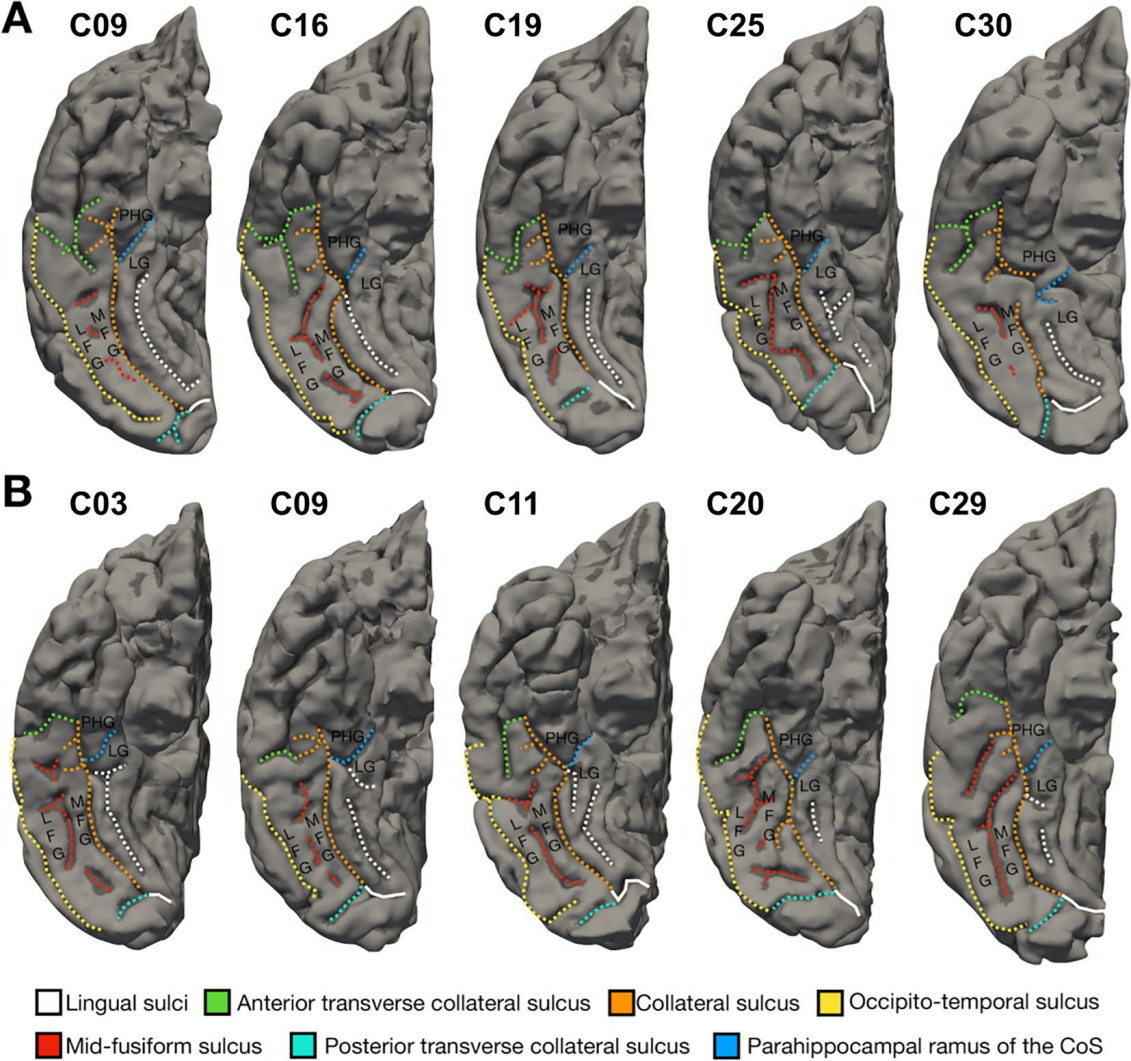
The mid-fusiform sulcus (MFS): a landmark dividing the lateral and medial fusiform gyrus (FG) in individual chimpanzee hemispheres. The MFS (red) is identifiable in all 60 hemispheres examined in the present study and is also a landmark that divides the lateral FG from the medial FG. (**A**) Cortical surface reconstructions of the right hemisphere from 5 different chimpanzees. (**B**) Cortical surface reconstructions of the left hemisphere from 5 different chimpanzees. To illustrate the similarity between hemispheres, the left hemisphere has been mirrored to have a consistent orientation as the right hemisphere. See legend for precise mapping between each sulcus and each color. *LFG* lateral fusiform gyrus, *LG* lingual gyrus, *MFG* medial fusiform gyrus, *PHG* parahippocampal gyrus.

Our first analysis examined if the MFS is identifiable in individual hemispheres of chimpanzee brains. Our approach revealed that the MFS was identifiable in all 60 hemispheres we examined with inter-individual variability among chimpanzees. As in humans, the MFS is best described as a longitudinal sulcus that divides the FG into lateral and medial partitions. In Fig. 1, we show five examples of MFS variability in right hemispheres (Fig. 1A) and left hemispheres (Fig. 1B) with the MFS labeled relative to the collateral (CoS) and occipito-temporal (OTS) sulci, which bound the FG medially and laterally, respectively. As illustrated in previous work in humans^25^, despite the fact that the MFS is present in each hemisphere, it varies in its fractionation and length. For example, the MFS can be a single longitudinal sulcus (C25, top row), fractionated into separate long and short sulcal components (C3, bottom row), or even fractionated into separate small sulci (C9, top row). We next quantified morphological features of VTC sulci in order to analyze similarities and differences in sulcal morphology between chimpanzees and humans.

### The average sulcal depth of the MFS is shallower than the OTS and CoS in both chimpanzees and humans

**Figure 2:**
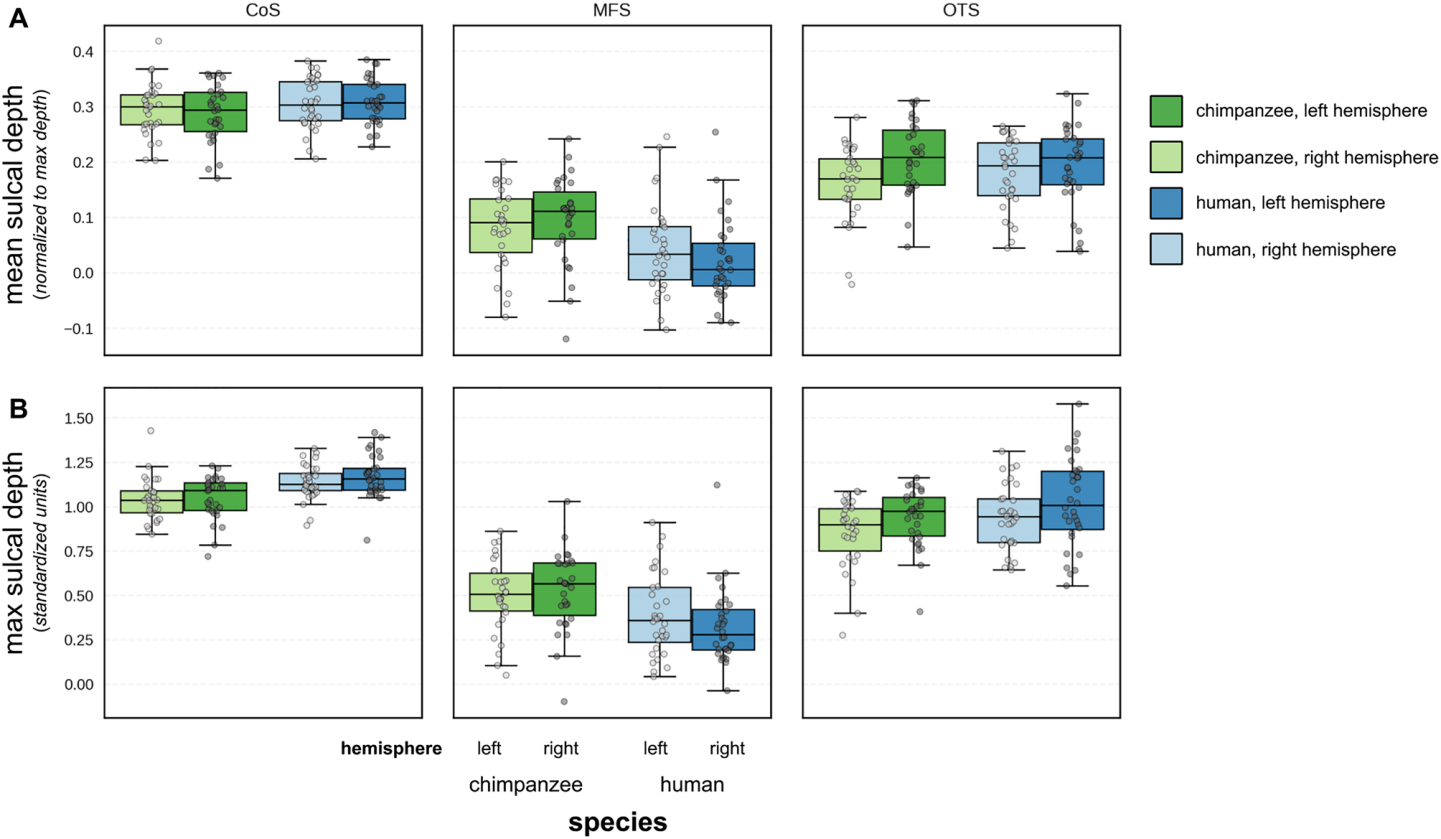
The MFS is relatively deeper in chimpanzees compared to humans. (**A**) Box plots indicating the median (± quartile) normalized sulcal depth for the collateral sulcus (CoS; left), mid-fusiform sulcus (MFS; middle), and the occipito-temporal sulcus (OTS; right) for the left (darker shade) and right (lighter shade) hemispheres in humans (blue) and chimpanzees (green). (**B**) Same layout, but for max sulcal depth (i.e., the sulcal pit in each sulcus). For both the mean and max normalized sulcal depth, the MFS is relatively deeper in chimpanzees than in humans.

As prior work in humans^26,29^ indicated that the most stable morphological feature of the MFS is its shallowness relative to the OTS and CoS, we first quantified the average sulcal depth (normalized to the max depth in each hemisphere, see “Materials and methods”) of the MFS, OTS, and CoS in humans and chimpanzees, which resulted in three main findings (Fig. 2A). First, a 3-way ANOVA (with sphericity correction) with species, sulcus, and hemisphere as factors found a main effect of sulcus (F(1.73,103.67) = 272.93, *p* < 0.0001; generalized eta squared (ges) = 0.70), in which the MFS was shallower than the surrounding OTS (*p* < 0.0001) and CoS (*p* < 0.0001) across species. Second, there were no differences regarding the average depth of the CoS or OTS across species (CoS, chimpanzees = 0.291 ± 0.05; CoS, humans = 0.307 ± 0.04, *p* = 0.234; OTS, chimpanzees = 0.184 ± 0.07; OTS, humans = 0.185 ± 0.07, *p* = 0.9115). Third, contrary to the stableness of the CoS and OTS depth across species, a species by sulcus interaction revealed that the MFS was relatively deeper in chimpanzees than humans (F(1.73,103.67) = 7.06, *p* = 0.002 (ges = 0.06); MFS, chimpanzees = 0.088 ± 0.07; MFS, humans = 0.03 ± 0.07, *p* < 0.001). When using the unnormalized, raw FreeSurfer depth values or a different normalization metric (gray matter volume), the MFS was also relatively deeper in chimpanzees than humans (*p* < 0.001; SI, Fig. 1).

### The sulcal pit of the MFS is deeper in chimpanzees compared to humans, while the opposite is true for the sulcal pit of the OTS and CoS across species

The deepest points of sulci, known as sulcal roots or sulcal pits, are believed to be the first points to form in development and under different levels of genetic control^33–35^. Interestingly, recent findings illustrate differences in the depth of sulcal pits, as well as their hemispheric asymmetry, between chimpanzees and humans in dorsal aspects of the temporal lobe^34^. Here, we tested if our previous analyses focusing on the mean sulcal depth also generalize to the sulcal pit. Interestingly, similar to the mean sulcal depth analyses, a 3-way ANOVA with species, sulcal pit (OTS/MFS/CoS), and hemisphere as factors revealed (a) a main effect of sulcus, in which the MFS pit was also relatively shallower than the OTS and CoS pit in both chimpanzees and humans [F(1.72,103.43) = 287.66, *p* < 0.0001 (ges = 0.71)] and (b) a species × sulcus interaction in which the MFS pit was relatively deeper in chimpanzees than humans (F(1.72,103.43) = 13.24, *p* < 0.0001 (ges = 0.10); MFS, chimpanzees = 0.51 ± 0.21; MFS, humans = 0.36 ± 0.22, *p* < 0.001). Nevertheless, post-hoc tests revealed a key difference between analyses comparing either the relative mean sulcal depth or the relative sulcal pit depth of the OTS and CoS between species: at the mean level, only the MFS, not the OTS or CoS differed across species (Fig. 2A), while at the sulcal pit level, the OTS and CoS were significantly deeper in humans compared to chimpanzees (CoS, chimpanzees = 1.05 ± 0.12; CoS, humans = 1.15 ± 0.11, *p* = 0.0038; OTS, chimpanzees = 0.89 ± 0.18; OTS, humans = 0.98 ± 0.22, *p* = 0.0191; Fig. 2B). Thus, while the normalized MFS pit is deeper in chimpanzees compared to humans, the opposite is true for the surrounding CoS and OTS pits, which are deeper in humans compared to chimpanzees. Taken together, these results indicate that the shallowness of the MFS relative to surrounding sulci in VTC is an evolutionarily preserved cortical feature, while the relationship of the sulcal pit depth among the three VTC sulci varies across species. We elaborate further on this sulcal depth patterning in the “Discussion” in relation to a theory of cortical folding known as *compensation*^34,35^.

### The MFS has a relatively thinner cortex in chimpanzees compared to humans

**Figure 3:**
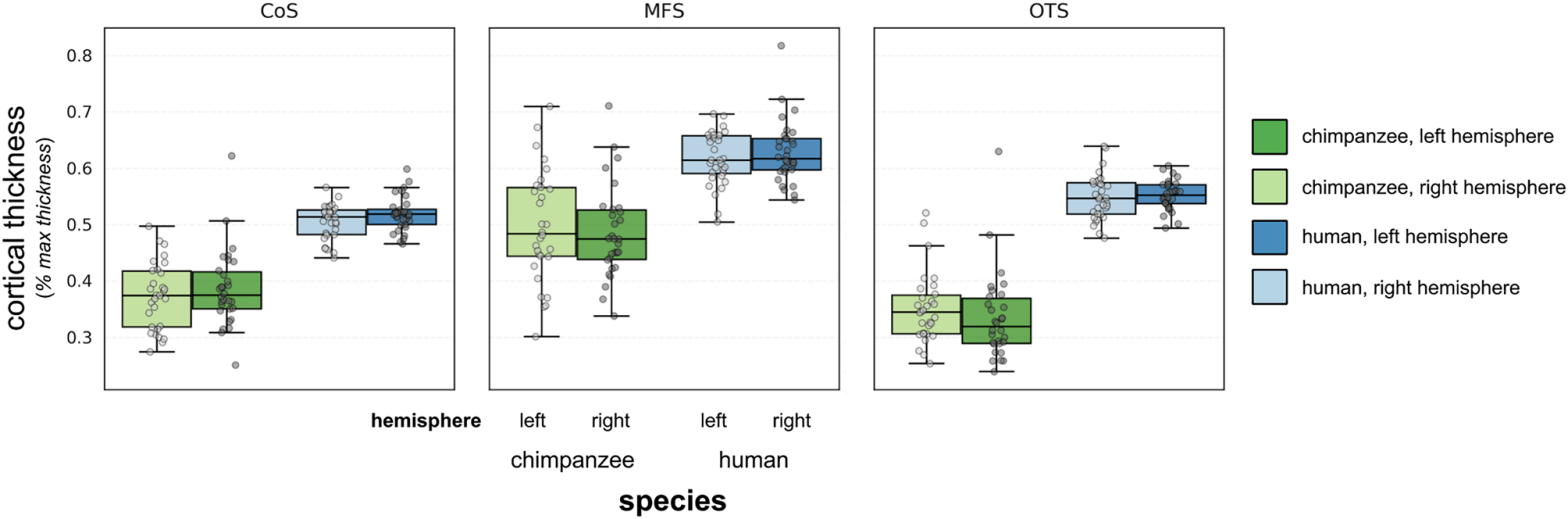
Sulci in ventral temporal cortex are relatively thinner in chimpanzees compared to humans. Box plots indicating the median (± quartile) cortical thickness (normalized to max thickness) for CoS (left), MFS (middle), and OTS (right) for the left (darker shade) and right hemispheres (light shade) in humans (blue) and chimpanzees (green). The CoS, MFS, and OTS are all relatively thinner in chimpanzees compared to humans.

We next compared the gray matter cortical thickness (normalized to the max thickness within each hemisphere) between chimpanzees and humans for the OTS, MFS, and CoS. A 3-way ANOVA with species, sulcus (OTS/MFS/CoS), and hemisphere as factors revealed a main effect of sulcus [*F*(1.95,117.13) = 173.45, *p* < 0.0001 (ges (0.64)]. In both chimpanzees and humans, the MFS was cortically thicker than the CoS and the OTS (MFS = 0.55 ± 0.10; CoS = 0.44 ± 0.08; OTS = 0.45 ± 0.12; Fig. 3). Interestingly, we also observed a main effect of species [*F*(1,60) = 258.95, *p* < 0.0001 (ges = 0.64)], in which the MFS was cortically thinner in chimpanzees compared to humans (MFS, chimpanzees = 0.49 ± 0.09; MFS, humans = 0.62 ± 0.05, *p* < 0.001). This effect also generalized to surrounding sulci, in which the OTS and CoS were also thinner in chimpanzees compared to humans (CoS, chimpanzees = 0.38 ± 0.06; CoS, humans = 0.51 ± 0.03, *p* < 0.0001; OTS, chimpanzees = 0.34 ± 0.07; OTS, humans = 0.55 ± 0.03, *p* < 0.0001; Fig. 3). When using the raw FreeSurfer thickness values (not normalized) or a different normalization metric (gray matter volume), there were still main effects of species [*F*(1,60) = 258.97, *p* < 0.0001 (ges = 0.64); *F*(1,59) = 63.6, *p* < 0.0001 (ges = 0.37)], in which sulci were relatively thicker in humans compared to chimpanzees (Supp. Fig. 2). Combined with our previous research showing that the FG is relatively thinner in chimpanzees than humans^36^, these findings show that a general feature of macroanatomical structures in high-level visual cortex of chimpanzees is their normalized thinness compared to homologous structures in human high-level visual cortex.

### Extensive variability in MFS length between chimpanzees and humans

**Figure 4:**
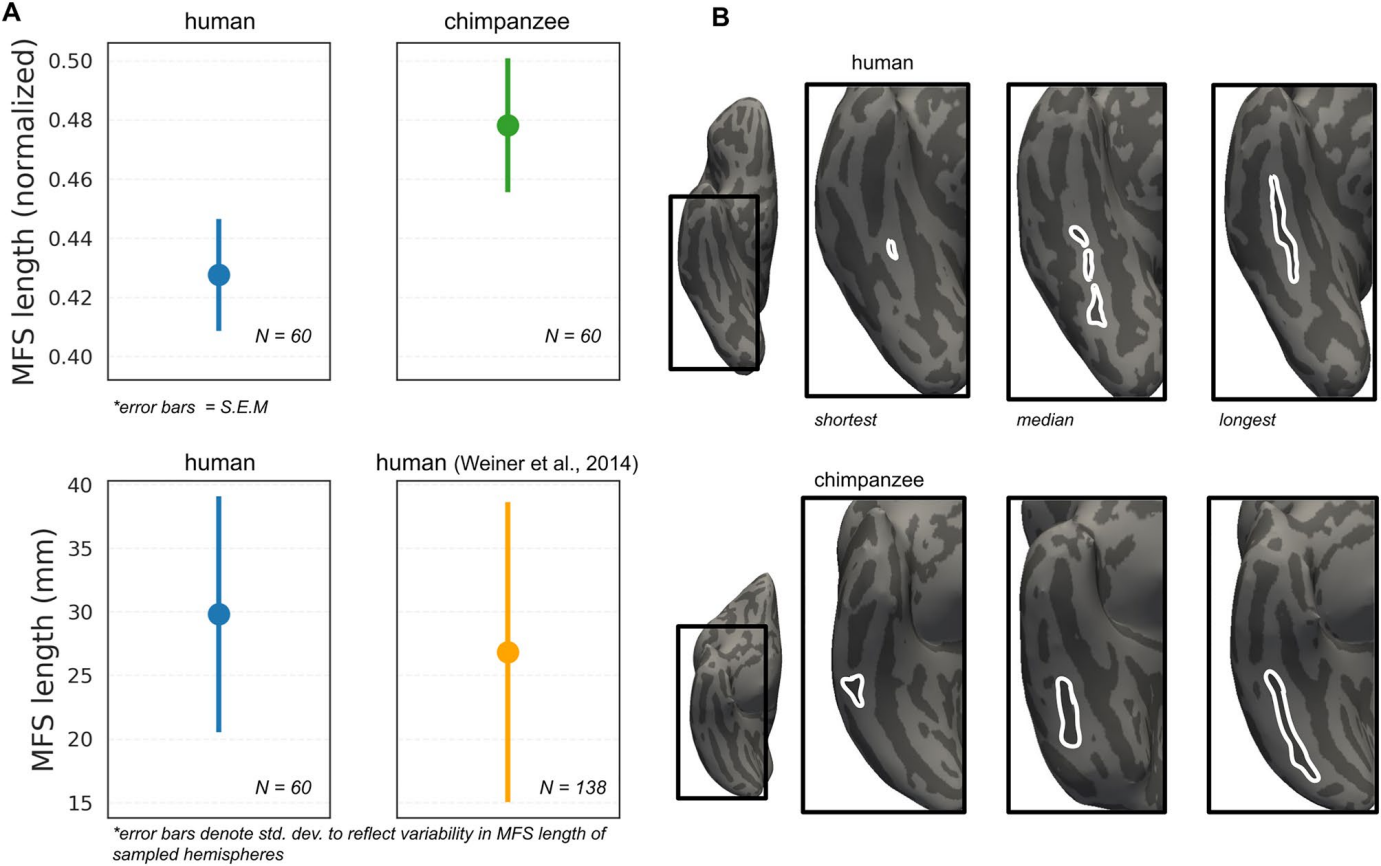
Comparison of MFS length across species. (**A**) Top, MFS length normalized to the length of the fusiform gyrus. There is no statistical difference in relative MFS length across species (MFS, chimpanzee = 0.48 ± 0.17, human = 0.42 ± 0.15 (% Fusiform gyrus length), *p* = 0.129). Bottom, comparison of MFS length (mm) variability in the present HCP sample and a previously reported sample (Weiner et al. 2014). Mean and variance of samples are similar despite differing methodology in length measurement (see “Materials and methods”). (**B**) Three right hemispheres from human (top) and chimpanzee (bottom) brains with the shortest (left), median (middle), and longest (right) MFS length in the present sample.

Previous findings have shown that the length of the MFS was its most variable feature in humans^29^. Specifically, the MFS could be as long as ~ 5.5 cm or as short as just over 2 mm. To test if the variability in MFS length across individuals and hemispheres was specific to humans, we measured the length of the MFS in chimpanzees. Our measurements revealed that there is also extensive variability in MFS length in chimpanzees relative to the length of the fusiform gyrus (see “Materials and methods”). Interestingly, there was no difference in relative MFS length across species (MFS, chimpanzee = 0.48 ± 0.17, human = 0.42 ± 0.15 (% Fusiform gyrus length), *p* = 0.1297; Fig. 4). Additionally, we emphasize the consistency of this effect across studies. Specifically, the raw average MFS length (mm) from the present group of 30 human participants (29.81 ± 9.36 mm) is close (both in mean and standard deviation) to our previous measurements from an independent group of 69 human participants across hemispheres (27.3 ± 10.8 mm; Fig. 4A).

### Predicting sulci in individual chimpanzees from a human template is possible for some, but not all, sulci in VTC

We further tested how well the MFS could be defined in an individual hemisphere by an independent definition provided by either an average chimpanzee cortical surface or an average human cortical surface. To do so, we first generated an average chimpanzee cortical surface from an independent set of 30 chimpanzee brains (which we refer to as the *chimp30* surface; see “Materials and methods”) and then calculated the similarity (DICE coefficient) between the MFS on the average cortical surface and the MFS defined within each individual. After aligning each individual chimpanzee and human cortical surface to (a) our average *chimp30* surface and (b) the average human cortical surface provided by FreeSurfer (fsaverage; https://www.freesurfer.net^37,38^), we then performed this calculation two additional ways: we compared the ability of the MFS defined on the *fsaverage* surface to predict the MFS defined on cortical surfaces from (1) 30 humans and (2) 30 chimpanzees. We then compared this prediction performance relative to prediction performances for the OTS and CoS.

**Figure 5:**
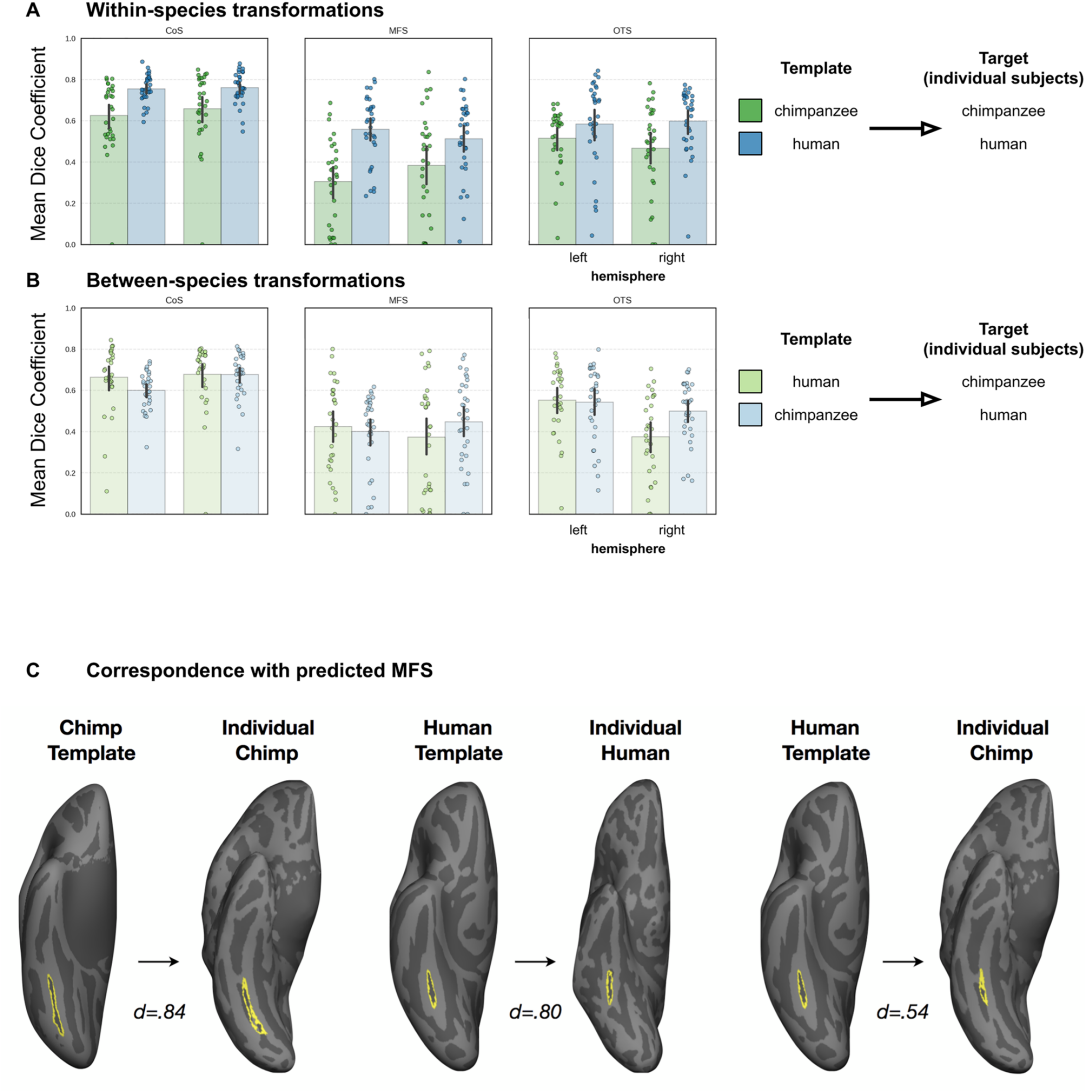
VTC sulci in chimpanzees can be predicted using sulcal definitions from human VTC. (**A**) Mean performance (as measured by the dice coefficient) to predict the CoS, OTS, and MFS within species. The three sulci were defined on the FreeSurfer average surface (human template), as well as an average chimpanzee surface that was generated from an independent set of 30 chimpanzee brains (*chimp30* template). Using cortex-based alignment, each sulcus was projected to each individual human and chimpanzee surface, which we refer to as the *predicted* sulcus. The alignment between the *predicted* and *actual* sulci (which were manually defined in each individual) was then quantified using the dice coefficient (“Materials and methods”). Green bars illustrate performance when using sulci defined on a chimpanzee template to predict sulci in individual chimpanzee cortical surfaces. Blue bars illustrate performance when using sulci defined on a human template to predict sulci in individual human cortical surfaces. In both species, prediction performance is highest for the CoS. Between species, there is better prediction performance in humans compared to chimpanzees. (**B**) Same as in (**A**) except sulci are predicted between species. Specifically, green bars illustrate performance when using sulci defined on a human template to predict sulci in individual chimpanzee cortical surfaces, while blue bars illustrate performance when using sulci defined on a chimpanzee template to predict sulci in individual human cortical surfaces. Prediction performance is highest for the CoS. Additionally, prediction performance in humans is higher when using a human compared to chimpanzee template for all sulci, while the species template used to predict VTC sulci does not affect performance when predicting the CoS across hemispheres and the right MFS in individual chimpanzee surfaces. (**C**) To give the reader a sense as to what higher (~ 0.8) vs. lower (~ 0.5) dice coefficients look like in terms of the correspondence between the predicted MFS on the cortical surface, three examples are included. *From left to right:* (1) predicting the MFS (yellow outline) in an individual chimpanzee using a chimpanzee template, (2) predicting the MFS in an individual human using a human template, and (3) predicting the MFS in an individual chimpanzee using a human template. The same individual chimpanzee cortical surface is included in the first and third column. In the former case, the chimpanzee template well-predicted the MFS in the individual chimp (leftmost column), while in the latter case, the MFS prediction from the human template is shifted medially, resulting in a lower dice coefficient (right column).

This approach resulted in three main findings. First, prediction performance varied by sulcus, with variability reflecting the appearance of each sulcus in development. Specifically, of these three sulci, the CoS appears first during fetal stages^39,40^ and has the highest predictability across participants and species, while the MFS appears last and has the lowest predictability (Fig. 5A, top). Second, prediction performance in humans (blue in Fig. 5A) is higher than in chimpanzees (green in Fig. 5A). Third, the ability for sulci defined in humans to predict sulci in individual chimpanzees varies by sulcus and hemisphere (3-way ANOVA with species, sulcus, and hemisphere as factors; sulcus × hemisphere interaction: *F*(1.81,108.31) = 12.09, *p* < 0.001 (ges = 0.03); Fig. 5A, bottom) as well as by species and hemisphere (species × hemisphere interaction: *F*(1,60) = 6.58, *p* < 0.01 (ges = 0.02)). For example, when predicting the location of the left or right CoS between species (Fig. 5B), there was no difference when using a cortical surface template built from either chimpanzees or humans (*p* = 0.162; *p* = 0.964; pooled post-hoc *t* tests), but there was a difference when predicting the right OTS (*t*(249) = 2.71, *p* = 0.007). To illustrate how a particular dice coefficient relates to sulcal definitions in VTC, we have included within- and between-species predictions in Fig. 5B. As hemispheric asymmetries vary by sulcus, we refrain from drawing conclusions regarding general hemispheric asymmetries between species. Rather, there may be more specific patterns of asymmetry, with the strongest difference between species in the right CoS. Additionally, even though some between-species predictions perform better than within-species predictions for a subset of sulci, we refrain from drawing widespread conclusions considering the rather small effect size. Together, these results reveal that predicting sulci in individual chimpanzees from a human cortical surface template is possible for some, but not all, sulci in VTC, with the most conserved predictions across species in the CoS.

### The MFS is also identifiable in bonobos, gorillas, orangutans, and gibbons

**Figure 6:**
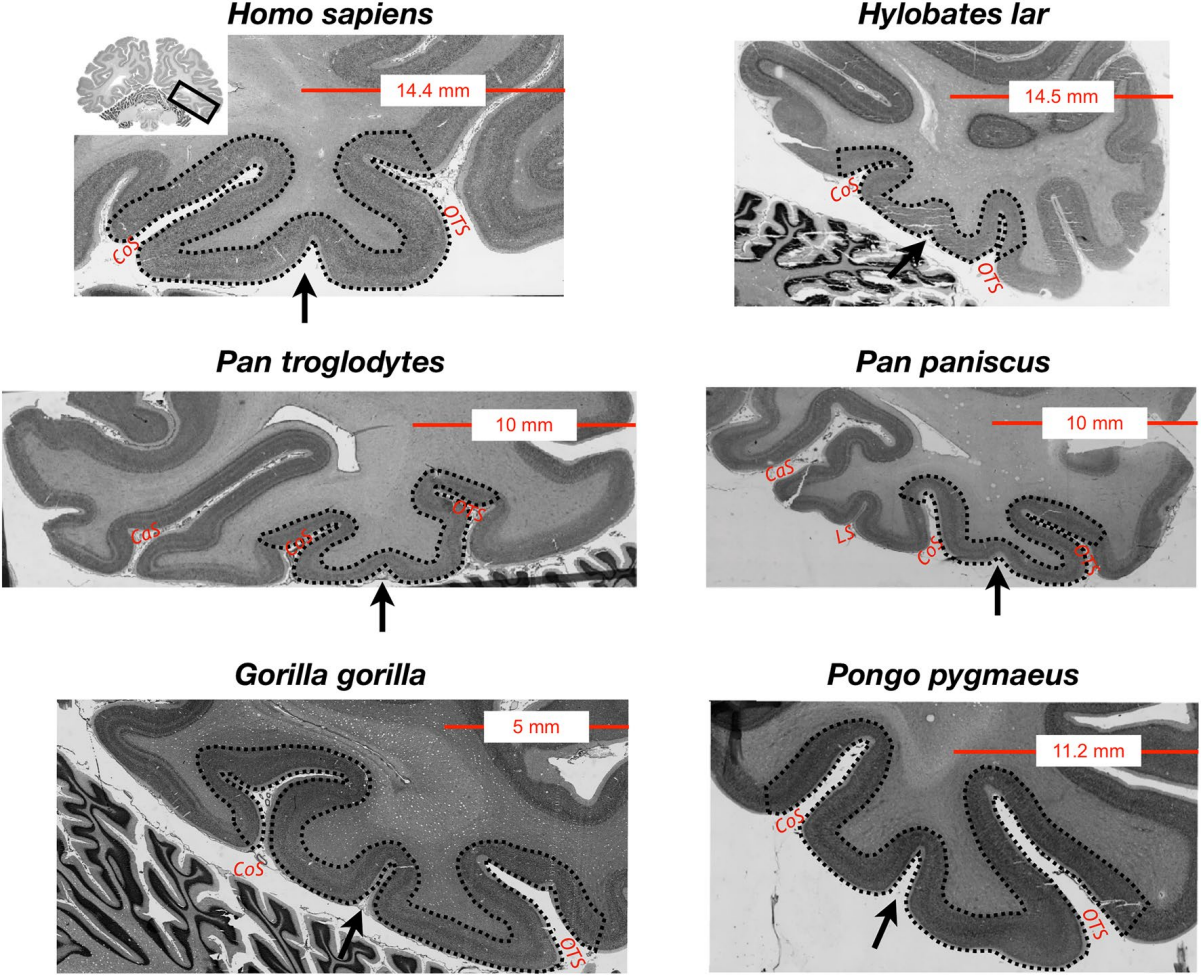
The mid-fusiform sulcus in the hominoid brain. The shallowness of the MFS relative to surrounding sulci (CoS, OTS) generates a distinctive pattern on histological coronal sections. We leveraged this fact to identify the MFS in not only humans and chimpanzees as in the prior analyses, but also additional hominoids (bonobos, gorillas, orangutans, and gibbons). Top left: Single histological section from a human brain sectioned in the coronal plane and stained for cell bodies using the Merker method^36^. Zoomed portion of the section at left within the black rectangle. Arrow: MFS. Additional histological coronal sections stained for cell bodies from the brains of a chimpanzee (*Pan troglodytes*), gorilla (*Gorilla gorilla*), gibbon (*Hylobates lar*), bonobo (*Pan paniscus*), and orangutan (*Pongo pygmaeus*). In each species, a similar pattern is generated in which the MFS is the shallow, intrafusiform sulcus surrounded by the deeper sulci laterally (OTS) and medially (CoS). The shallow intralingual sulcus (LS) is also visible in the histological slice of the bonobo (*Pan paniscus*; middle right).

**Figure 7:**
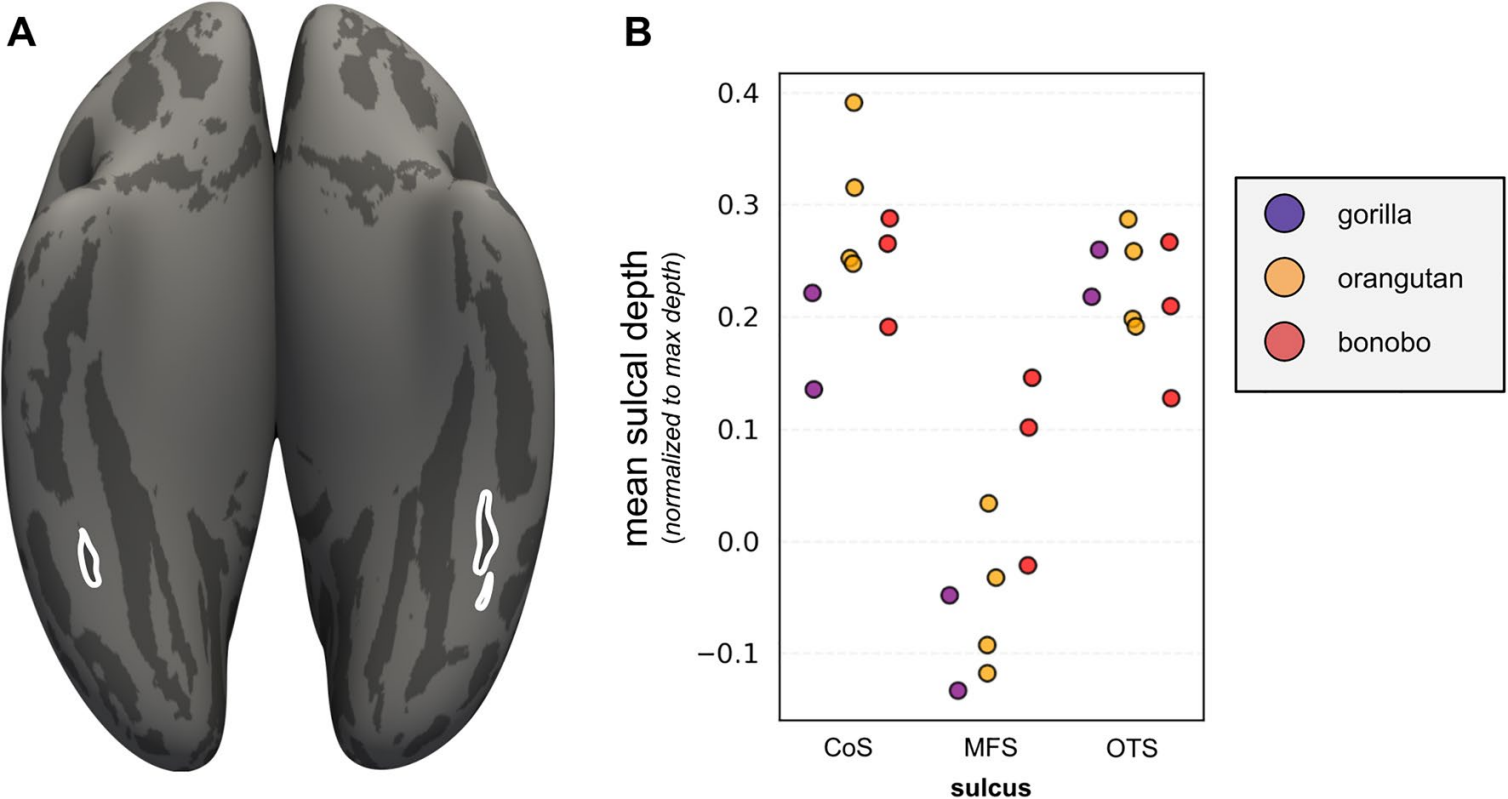
Depth patterns of the CoS, MFS, and OTS in gorilla, orangutan, and bonobo. (**A**) Example inflated cortical surface reconstruction from an orangutan brain with the MFS outlined in white. (**B**) Strip plots indicating the ratio of normalized mean sulcal depth in the CoS (left), MFS (middle), and OTS (right) in gorilla (N = 2 hemispheres), orangutan (N = 4 hemispheres), and bonobo (N = 3 hemispheres). The MFS was shallower than the surrounding CoS and OTS. Due to the large differences in sample sizes, we only statistically compare between-species differences between humans and chimpanzees in which sample sizes are (a) large and (b) matched between species.

Prior work^26^ indicated that the shallowness of the MFS generates a distinctive pattern relative to surrounding sulci in coronal histological sections of the human brain. In Figs. 6 and 7, we show that the MFS is identifiable in histological sections^41^ and cortical surface reconstructions in other hominoids such as bonobos, gorillas, orangutans, and gibbons. In SI Figs. S4–S8, we also include the identification of the MFS relative to the OTS and CoS among histological sections of each species. As the OTS and CoS are over twice as deep as the MFS, the relationship among the depths of these three sulci generates an omega-like pattern in single coronal histological sections. As illustrated in Fig. 6, this can also be observed in coronal histological sections from the brains of chimpanzees (*Pan troglodytes*), bonobos (*Pan paniscus*), gorillas (*Gorilla gorilla*), orangutans (*Pongo pygmaeus*), and gibbons (*Hylobates lar*). In each hominoid brain—whether from a human or non-human—the relationship among these three sulci generates this omega-like pattern, in which the MFS is the shallower sulcus between the deep OTS and CoS (Fig. 6). To demonstrate that this pattern is represented along the length of the MFS, we show several histological sections from each post-mortem sample in Figures S4–S8. To explore this further, in a smaller subset of orangutan (N = 4 hemispheres), bonobo (N = 3 hemispheres), and gorilla (N = 2 hemispheres) from previously published work^4^, we measured the depth of the MFS, CoS, and OTS on cortical surface reconstructions as we did for chimpanzees and humans (Fig. 7). Similar to chimpanzees and humans, the MFS is shallower than the OTS and CoS across species (Fig. 7). However, while the MFS is relatively deeper in chimpanzees compared to humans (Figs. 2 and 6), the MFS is not always deeper in non-human hominoids compared to humans (Fig. 7). For example, the MFS in the gibbon is very shallow and appears more like a dimple. Due to the small sample sizes, we refrain from running statistics across all six species. Altogether, the stable morphological feature of the MFS across species is its shallowness relative to the OTS and CoS. Additionally, there is variability among species with regard to the shallowness of the MFS.

## Discussion

We identified and compared morphological features of the mid-fusiform sulcus (MFS) relative to surrounding sulci in ventral temporal cortex (VTC) between humans and other hominoid primates for the first time, with an extended analysis comparing humans and chimpanzees. Across species, the MFS is especially stable in its shallowness relative to surrounding VTC sulci and variable in its length. Interestingly, while the MFS is relatively deeper and the cortex of the MFS is relatively thinner in chimpanzees compared to humans, its topological position within the FG (after scaling the chimpanzee brain) is so similar between species that implementing cortex-based alignment across species reveals that the location of some sulci within VTC (CoS in both hemispheres and the MFS in the right hemisphere) are predicted equally well in chimpanzees using either a human or chimpanzee template. Finally, our analyses showed that the MFS is not only present in humans and chimpanzees, but also in other apes—bonobos, gorillas, orangutans, and gibbons. Taken together, these results reveal that the MFS is not just a human-specific landmark in VTC, but a sulcal landmark that is a shared feature of hominoid brains in which the MFS divides the FG into lateral and medial partitions. We discuss these results in the context of (a) functional and cytoarchitectonic maps within high-level visual cortex, (b) the sulcal patterning of VTC in hominoids, and (c) the compensation theory of cortical folding.

### The MFS: a functional and cytoarchitectonic landmark across species?

Within the last decade, a number of studies in humans have shown that the MFS is a useful landmark identifying functional and cytoarchitectonic transitions in VTC. Specifically, the MFS identifies transitions in large-scale functional gradients (or maps) that span ~ 5000 mm^3^^14,42–46^ and fine-scale functional regions, which are approximately an order of magnitude smaller. Furthermore, many different functional maps in VTC are organized along a lateral-medial anatomical dimension and the entire MFS identifies functional transitions in these many maps^11^. Finally, merely identifying the anterior tip of the MFS identifies a region that is selective for images of faces^29,30^ that is causally implicated in face perception^16,17^.

A handful of recent studies of human brains also show that like the large-scale functional gradients in VTC, microstructural gradients are also organized along a lateral-medial macroanatomical dimension. Specifically, using observer-independent methods that are blind to cortical folding, these studies showed that cytoarchitectonic transitions among four areas of the FG occur within the MFS in which two areas (FG1 and FG3) are situated medial to the MFS and two other areas (FG2 and FG4) are situated lateral to the MFS^29,30,47^. An additional study revealed that the MFS also marks differences in receptor density across cortical layers (known as receptor architecture) in the posterior FG (i.e., the border between areas FG1 and FG2^48^), which has functional implications for interpreting cellular architecture as receptors are key molecules of neurotransmission. Taken together, these findings show that identifying the MFS on the cortical surface has predictive power in providing cellular-scale insight regarding the cortical layout of large-scale functional maps and fine-scale functional regions in human VTC.

**Figure 8:**
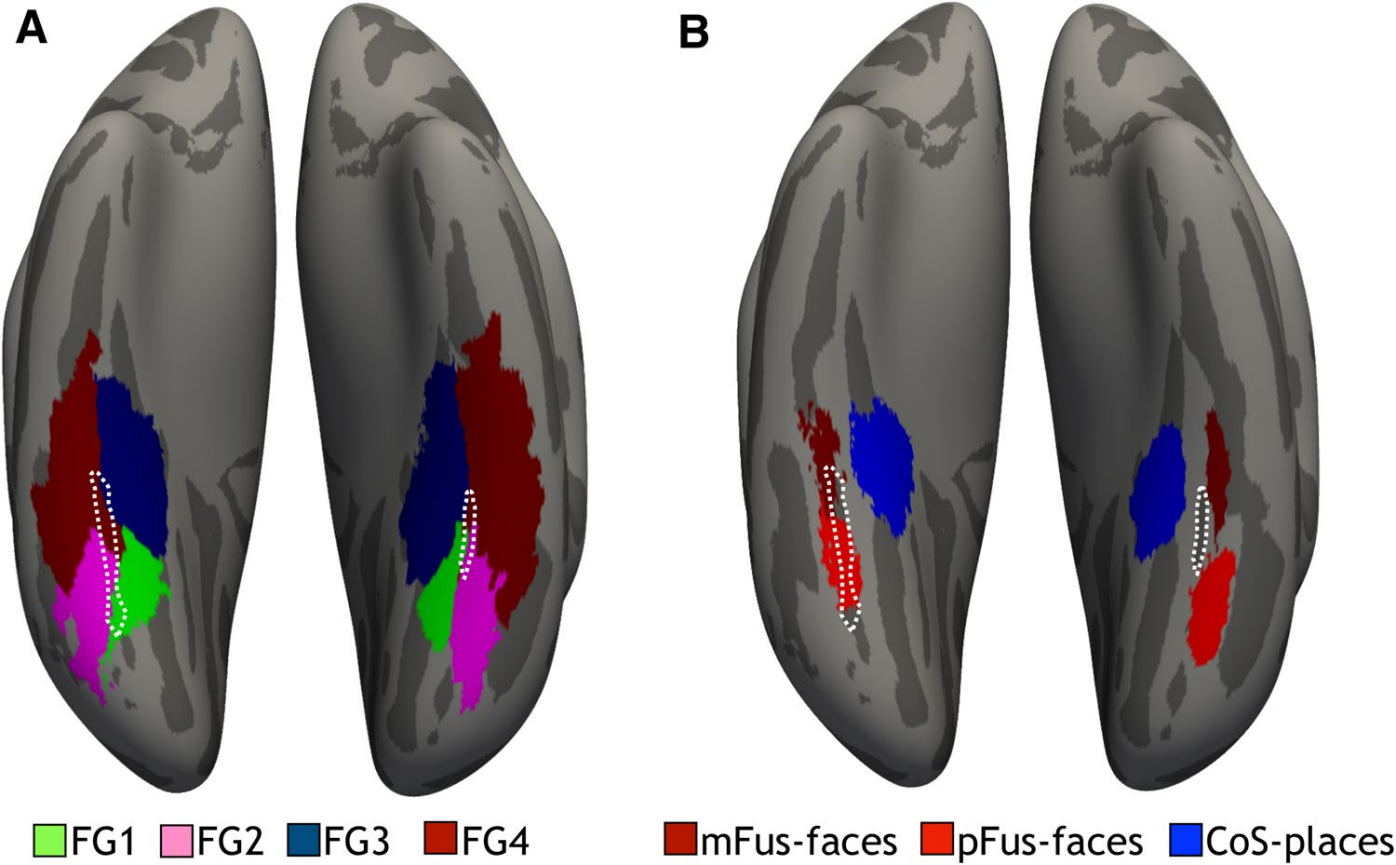
The MFS: A landmark identifying microarchitectonic and functional transitions in VTC of non-human hominoids? (**A**) Using cortex-based alignment (CBA), the probabilistic cortical location of four cytoarchitectonic areas (across 10 individuals; from Caspers et al. 2012; Weiner et al. 2014, 2017; Lorenz et al. 2015) within the human FG (areas FG1, FG2, FG3, and FG4) were projected to an average of 30 chimpanzee cortical surfaces. (**B**) Using CBA, the probabilistic cortical location of face- (mFus-faces and pFus-faces) and place-selective (CoS-places) regions from 12 individuals (from Weiner et al. 2017) were projected to the same average cortical surface as in (**A**). (**A**,**B**) are cytoarchitectonic and functional parcellations of chimpanzee VTC predicted by cytoarchitectonic and functional parcellations of human VTC, respectively. Future studies can leverage the fact that the MFS predicts the location of cytoarchitectonic and functional regions in humans to directly compare micro- and macroanatomical features of these areas between species. *CoS* collateral sulcus, *FG* fusiform gyrus, *mFus* mid-fusiform, *pFus* posterior fusiform. Dotted white line: mid-fusiform sulcus.

Is the MFS also a landmark identifying functional and microarchitectonic transitions in VTC of non-human hominoids? Since ethical and practical restrictions limit the use of invasive methods to study ape brain structure and function, answering this question regarding functional transitions will be difficult. Nevertheless, using positron emission tomography, a previous study revealed that like humans, chimpanzees have face-selective activations on the FG^5^. Because the MFS is topologically preserved across species as we show in the present study and face-selective regions are so tightly coupled with the MFS in humans^14,26,30,44,49^, it is likely that face-selective regions are also tightly coupled with the MFS in other hominoids. Importantly, future parcellation of the FG with respect to the MFS across hominoids is feasible using microarchitectonic data such as myeloarchitecture^50^ or cytoarchitecture^51–53^. For example, as previous studies identified the MFS as a cytoarchitectonic landmark in humans^26^, the same observer-independent approach could be implemented using histological sections in non-human hominoids (Fig. 8). As cytoarchitectonic features differ in the FG between chimpanzees and humans^54^, an interesting option is that the MFS also identifies cytoarchitectonic transitions in other hominoids, but cytoarchitectonic features of areas within the FG might differ between species. Finally, as non-invasive tools are available that are related to myelin content (the ratio between T1-weighted and T2-weighted scans using magnetic resonance imaging, for example^55^) and these tools reveal a lateral-medial gradient in human VTC in which a transition in this gradient is predicted by the MFS^14,56^, the correspondence between the MFS and this T1/T2 gradient can also be examined in chimpanzees using publicly available data (https://www.chimpanzeebrain.org/) in future studies. These future comparisons can use cortex-based alignment to quantify the relationship between parcellations of chimpanzee VTC predicted by cytoarchitectonic and functional parcellations of human VTC, respectively (Fig. 8).

### Reconsidering the sulcal patterning in ventral occipito-temporal cortex in hominoids

Historically, anatomists had a difficult time characterizing the sulcal pattern in ventral occipito-temporal cortex in chimpanzees^34,57–59^. Here, we have proposed (see Appendix) that there are at least five underlying reasons contributing to this difficulty: (1) gyri and sulci surrounding the MFS were accurately labeled, while the MFS was depicted and unlabeled^57^, (2) gyri were accurately identified, but sulci were mischaracterized^58^, (3) the MFS was present and unlabeled in photographs and images, while the fusiform is mis-localized, (4) the MFS was mentioned, but not in the context of the human or chimpanzee brain^34^, and (5) the MFS is not mentioned and while the fusiform is discussed briefly, it is obscured by the cerebellum in photographs^59^. Despite these five issues, when revisiting the sulcal patterning of ventral occipito-temporal cortex of non-human hominoids while also considering the MFS in (1) in vivo T1 MRI scans (present study), (2) postmortem histological tissue (present study), and (3) the plates and figures from these classic studies as illustrated in the Appendix, sulcal definitions in non-human hominoids are more consistent than reported by these classic studies and very similar to sulcal definitions in humans^26^ as reported in the present study.

**Figure 9:**
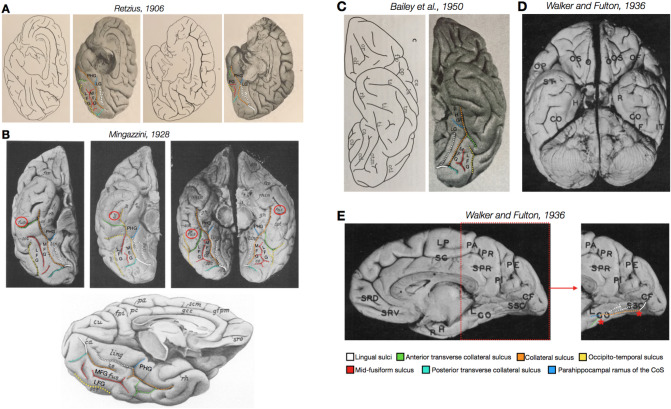
Reconsidering the sulcal patterning in classic studies of ventral occipito-temporal cortex in chimpanzee brains. (**A**) The medial surface of two chimpanzees (*Troglodytes niger*) from Retzius (1906). While Retzius only labeled the collateral (*co*) and calcarine (*ca*) sulci, each of the sulci that we identify in the present study is identifiable in Retzius’ images. We refer to this as a case in which the MFS is identifiable, but unlabeled. (**B**) *Top:* photographs of the ventral surface of four chimpanzee hemispheres (3 right and 1 left) from Mingazzini (1928). In each case, the fusiform (*fus*, red circle) is mis-labeled and is far too anterior considering modern definitions. Nevertheless, each of the sulci that we identify in the present dataset is identifiable in these images. Additionally, Connolly (1950) also previously commented on the labeling errors in Mingazzini’s (1928) paper (see text). We refer to this as a case in which the MFS is present and unlabeled, while the fusiform gyrus is mis-localized. *Bottom*: An additional left hemisphere provided by Mingazzini (1928), which (a) identifies the fusiform correctly and (b) only corresponds to the medial fusiform (MFG), while the MFS and lateral fusiform are unlabeled. (**C**) *Left:* a labelled schematic of the sulcal patterning within the ventral view of a chimpanzee left hemisphere. *Right*: the photograph that was used to generate the schematic depicted in the image at left by Bonin et al., 1950. Both the schematic and photograph were used as representative illustrations accompanying the discussion of sulcal patternings in the chimpanzee brain. It is worth noting that Bonin and colleagues mis-identified the collateral sulcus (*col*). Specifically, what is labeled *col* is actually the lingual sulcus, which is located medial to the collateral sulcus. Instead, what is labeled otm is actually the collateral sulcus (see text for further explanation). Further, the MFS is also clearly identifiable in addition to the other sulci identified in the present paper. We refer to this as a case in which gyri were accurately identified, but sulci were mischaracterized. Also, note some inaccuracies in the drawing compared to the photograph. For example, the MFS is inaccurately small in the drawn schematic compared to the photograph. (**D**) Ventral view of a photograph of a chimpanzee brain (“Bonzo”) from Walker and Fulton (1936). The anterior portion of the fusiform gyrus is labeled (**F**), but the rest of the fusiform is obscured. We consider this as a case in which the fusiform is discussed, but a majority of it is obscured by the cerebellum. (**E**) An additional photograph of a medial view of a chimpanzee right hemisphere (“Song”) from Walker and Fulton (1936). The collateral sulcus is correctly labeled and the visible sulcal patterning resembles that identified in the present study. Nevertheless, what we identify as the start and end of the MFS (red asterisks) cannot be differentiated from the OTS as this view is too medial. But, as the MFS is immediately lateral to the CoS in every hemisphere examined in both present and historical images, these asterisks almost certainly correspond to the anterior and posterior extents of the MFS. *LFG* lateral fusiform gyrus, *LG* lingual gyrus, *MFG* medial fusiform gyrus, *PHG* parahippocampal gyrus.

Given the historical difficulty in characterizing the sulcal pattern in VTC, one might ask what may have contributed to these difficulties, especially since Retzius was the first to identify the MFS in humans^57^, but did not define the MFS in chimpanzees in another atlas that he published a decade later despite the fact that the MFS is visible (but unlabeled) in his images (^60^; Fig. 9). In our prior work^61^, we have discussed the complications of discriminating shallow, tertiary sulci such as the MFS from artificial indentations produced by veins and arteries in postmortem specimens. Thus, we speculate that this issue contributed to the difficulty that these previous authors experienced in trying to make sense of the seeming complexity of the sulcal pattern in the ventral occipito-temporal lobe (Appendix). The quantifications made in the present work were conducted using sulcal definitions from cortical surface reconstructions that were made from the boundary between gray and white matter on the inner surface of the cerebrum, and thus, are not confused with indentations produced by veins and arteries on the outer surface of the cerebrum. This approach is in stark contrast to classic approaches and likely contributed to the ease in the identification of the MFS in the present work, as well as the direct alignment and quantification of the MFS between humans and chimpanzees. Together, these findings require a reconsideration of the sulcal patterning within ventral occipito-temporal cortex of ape brains that incorporates the MFS (Fig. 9).

### Modifying the theory of compensation: Local vs. average morphological features

What mechanisms may explain the fact that the MFS is relatively deeper and cortically thinner in chimpanzees than in humans? One might consider that *compensation* may explain the difference in depth in the MFS across species. Specifically, Connolly^34,35^, referred to *compensation* as a way to describe how sizes of sulci seemingly counterbalanced those of their neighbors. For instance, a shallow, short sulcus would neighbor a particularly long and deep sulcus in which the former would “compensate” for the latter and in turn, make the overall degree of cortical folding within a given region approximately equal^62^. Our results expand on and modify this proposal in two main ways. First, compensation may not always occur when considering the average depth of a sulcus. For instance, when considering the fact that the average sulcal depth of the MFS is relatively deeper in chimpanzees than humans, compensation would predict that the average depth of the CoS and OTS would then be deeper in humans compared to chimpanzees. However, this is not the case as there is no difference in the normalized average CoS or OTS depth between species (Fig. 3A). Second, compensation may occur on a local scale (i.e. gyral crown or sulcal pit) as opposed to the average morphological features of an entire macroanatomical structure (i.e. average depth of a sulcus or average height of a gyrus). Specifically, contrary to our measurements of the average sulcal depth, our measurements of sulcal pits for the MFS, OTS, and CoS do indeed support predictions of compensation. That is, the MFS pit is relatively deeper in chimpanzees compared to humans, while the CoS and OTS pits are relatively deeper in humans compared to chimpanzees (Fig. 3B). Thus, our results encourage future studies considering predictions of the compensation theory of cortical folding to also consider assessments of local morphological features in addition to the average morphological features of macroanatomical structures in their entirety.

## Conclusion

The human fusiform gyrus (FG) contains a tertiary, longitudinal sulcus known as the mid-fusiform sulcus (MFS) that bisects the FG into lateral and medial parallel gyri. The MFS is a functional and architectonic landmark in the human brain. Here, we show that the MFS is also identifiable in apes including chimpanzees, bonobos, gorillas, orangutans, and gibbons. Interestingly, the MFS is morphologically different across species. For example, the MFS is relatively deeper and cortically thinner in chimpanzees compared to humans. Taken together, these results reveal that the MFS is not only a human-specific cortical landmark, but also a sulcal landmark that is shared between humans and other hominoids.

## Materials and methods

### Participants

#### Human

We randomly selected 30 human participants (19 female; 11 male; ages between 22 and 36) from the database provided by the Human Connectome Project (HCP): https://www.humanconnectome.org/study/hcp-young-adult. HCP consortium data were previously acquired using protocols approved by the Washington University Institutional Review Board. As our previous morphological analyses of the MFS^29^ did not show any sex differences across a range of participant ages (from 5–85), we did not specifically balance sex when selecting participants. Moreover, the chimpanzee sample also contains a similar ratio of female to male participants.

#### Chimpanzee

Anatomical T1 scans were previously acquired using MRI in 60 chimpanzees (38 female; 22 male; ages between 9 and 54), and no new data were collected for the present study. The chimpanzees were all members of the colony housed at the Yerkes National Primate Research Center (YNPRC) of Emory University. All methods were carried out in accordance with YNPRC and Emory University’s Institutional Animal Care and Use Committee (IACUC) guidelines. Institutional approval was obtained prior to the onset of data collection. Further data collection details are described in Keller et al. 2009^63^. Chimpanzee MRIs were obtained from a data archive of scans collected prior to the 2015 implementation of U.S. Fish and Wildlife Service and National Institutes of Health regulations governing research with chimpanzees. These scans were made available through the National Chimpanzee Brain Resource (https://www.chimpanzeebrain.org; supported by NIH grant NS092988).

### Data acquisition

#### Humans

Anatomical T1-weighted MRI scans (0.8 mm voxel resolution) were obtained in native space from the HCP database, along with outputs from the HCP modified FreeSurfer pipeline (see^64^ for T1 and FreeSurfer pipeline details).

#### Chimpanzees

Detailed descriptions of the scanning parameters have been described in Keller et al. 2009^63^, but we describe the methods briefly here. Specifically, T1-weighted magnetization-prepared rapid-acquisition gradient echo (MPRAGE) MR images were obtained using a Siemens 3 T Trio MR system (TR = 2300 ms, TE = 4.4 ms, TI = 1100 ms, flip angle = 8, FOV = 200 mm × 200 mm) at YNPRC in Atlanta, Georgia. Before reconstructing the cortical surface, the T1 of each chimpanzee was scaled to the size of the human brain. As described in Hopkins et al. 2017^36^, within FSL, (1) the BET function was used to automatically strip away the skull, (2) the FAST function was used to correct for intensity variations due to magnetic susceptibility artifacts and radio frequency field inhomogeneities (i.e., bias field correction), and (3) the FLIRT function was used to normalize the isolated brain to the MNI152 template brain using a 7 degree of freedom transformation (i.e., three translations, three rotations, and one uniform scaling), which preserved the shape of individual brains. Next, each T1 was segmented using FreeSurfer. The fact that the brains are already isolated, both bias-field correction and size-normalization greatly assisted in segmenting the chimpanzee brain in FreeSurfer. Furthermore, the initial use of FSL also has the specific benefit, as mentioned above, of enabling the individual brains to be spatially normalised with preserved brain shape, and the values of this transformation matrix and the scaling factor were saved for later use.

### Analysis of morphological features

#### Labeling of sulcal landmarks

Manual identification was performed to identify three prominent sulci (CoS, MFS, OTS) in the ventral temporal cortex for each human and chimpanzee brain hemisphere, in accordance with the definitions outlined in^26,29^. Authors JAM, WV, and KSW defined these sulci. The surface vertices for each sulcus were selected using tools in FreeSurfer, and saved as surface labels for vertex-level analysis of morphological statistics.

#### Calculation of sulcal morphology

##### Sulcal depth and thickness

Mean and maximal depth were calculated for each sulcal label from the .sulc maps generated by FreeSurfer^38,65^. Because chimpanzee T1 MRIs were linearly scaled to human brain size to facilitate quantitative morphological algorithms to run appropriately, mean sulcal depth at each vertex was normalized to the maximal depth within each hemisphere for both species. Mean cortical thickness (in mm) was generated for each sulcus through the *mris_anatomical_stats* function in FreeSurfer and also scaled to the vertex of max thickness within each individual hemisphere. Both depth and thickness values were scaled to the max within each hemisphere because the chimpanzee anatomical MRIs were linearly scaled in order to facilitate cortical surface reconstruction. We also show that the demonstrated morphological patterns hold when using raw values from the FreeSurfer pipeline (without normalization to a max hemisphere), or with normalization to a different metric—total gray matter volume of each hemisphere (Supp. Fig. 1, Supp. Fig. 2).

##### Sulcal length

The length of each sulcus was calculated by obtaining the longest geodesic distance along the cortical surface between any pair of vertices on the border of the sulcal label. When sulci were identified as consisting of multiple disconnected pieces on the cortical surface, the sulcal length was defined as the total length of each sulcal component [not including the annectant gyral component(s)]. The length values were then normalized relative to the length of the fusiform gyrus of the same hemisphere for both chimpanzee and human brains. This approach produced sulcal length values (in mm) in accordance with previous reports in humans despite differing methodology (Fig. 4). Here, geodesic distance was calculated on the *fiducial* surface using algorithms implemented in the pycortex (https://gallantlab.github.io/) package^66^.

#### Within- and between-species transformations

To quantify the variability of sulcal locations within and between species, we used cortex-based alignment^37^ to transform human and chimpanzee template hemispheres onto target individual hemispheres. The template human surface was the *fsaverage* brain^37,38^, while we created a chimpanzee template surface from a held-out population of 30 chimpanzee brains with the FreeSurfer *make_average_subject* function. Similarity between each transformed template label and the target individual subject label was calculated at the vertex-level with the Dice coefficient, where X and Y are the vertex locations in each sulcus label:

$$DICE(X,\;Y) = \frac{{2\left| {X \cap Y} \right|}}{\left| X \right| + \left| Y \right|}$$

### Statistical methods

All repeated measures ANOVAs and post-hoc testing were performed with the *afex* and *emmeans* R packages, imported into Python via rpy2. For each ANOVA, cortical hemisphere and sulcus were used as within-subject factors, while species was implemented as a between-subject factor. The Greenhouse-Geisser sphericity correction method was applied for all ANOVAs, which adjusted the degrees of freedom. Effect sizes for each main effect and interaction were calculated and reported with the generalized eta squared metric. Post-hoc *t* tests were conducted as pooled *t* tests of contrasts for the ANOVA models, as implemented in the *emmeans* R package.

### Electronic supplementary material

Supplementary Figures.

## Appendix

Our present findings reveal that sulcal definitions within ventral temporal cortex (VTC) in non-human hominoids are more consistent than the variability that has been reported by classic studies. Consequently, we thought it would be useful to include images and text from foundational studies to relate to the present VTC sulcal definitions. In doing so, we provide clarity regarding the historical confusion of the sulcal pattern in VTC and show that the modern sulcal definitions implemented in the present study can also be applied to the brains included in the figures from these classic studies. Furthermore, and as included in the “Discussion”, we have determined (at least) five main reasons why the MFS has been missed in these previous studies (see Fig. 9 for accompanying images):

1. *Gyri and sulci surrounding the MFS were accurately labeled, while the MFS was depicted and unlabeled*. For example, consider images from Retzius (1906)^57,60^ in which just the collateral (*co*) and calcarine (*ca*) sulci were labeled in ventral occipito-temporal cortex. The MFS is also clearly depicted in both images, as well as in Retzius’ schematics.
2. *The MFS is present and unlabeled, while the fusiform is mis-localized*. Both Connolly (1950)^34^ and Bailey et al. (1950)^58^ credit Mingazzini's seminal work in 1928^67^ examining the sulcal patterning in 15 chimpanzee brains. And yet, when looking closely at Mingazzini's figures and plates, the FG is mis-localized. As depicted in Fig. 9, the reader can see that the FG (*fus*, red circle) is anterior to what modern definitions consider the anterior limit of the FG (the anterior transverse collateral sulcus, green). On the contrary, when considering the modern definition of the FG that we use throughout this paper, the MFS is easily identifiable (red dotted line). Interestingly, Connolly (1950) also mentions that Mingazzini's figures are inaccurate in the context of sulcal patterns within the frontal lobe. Connolly writes:
  "*It may be observed that mistakes in labeling occur in some of the figures, which might easily lead to confusion if the text were not carefully read. For example, in Mingazzini's figure 6 the sulcus sop is not the sulcus opercularis but the subcentral anterior, marked sba in his other figs. 27, 46." P. 11*.
3. *Gyri were accurately identified, but sulci were mischaracterized*. For example, in the widely cited work from Bailey et al. 1950, the collateral sulcus is mis-identified. As illustrated in the figures in Fig. 9, what is labeled 'col' is actually the lingual sulcus, which is (a) located medial to the collateral sulcus and (b) also unlabeled in the Retzius images. Instead, what is labeled *otm* is actually the collateral sulcus. Interestingly, the authors define the collateral and lateral occipitotemporal sulci in their explanation of the fissural pattern, but they do not include a description of what refers to their *otm* [Bailey et al. (1950) write of the confusion regarding the fissural pattern in the temporal lobe. The authors write: "*col, the collateral sulcus.* A fairly short sulcus, just below, and parallel to the calcarine fissure, has been termed the collateral for the purposes of orientation on our brain-map. The fissures in this region are so variable that no exact homologies can be instituted. The fissural pattern of the temporal lobe consists in all primates of a number of furrows, running approximately parallel to the Sylvian fissure. The number of these sulci varies with the size of the brain. When there are only three, it is customary to speak of a superior (or parallel), a middle, and an inferior temporal sulcus. If, as in larger primate brains, a fourth is present, it is here called the lateral occipitotemporal sulcus. Walker and Fulton call the inferior temporal and collateral fissure lateral and medial occipitotemporal sulcus, respectively. But this is only a difference in nomenclature." (P. 31)]. Instead, they only explain how Walker and Fulton (1936)^59^ use the term otm to refer to the collateral. What is perhaps most relevant for the present discussion is that in the beginning of their explanation, Bailey and colleagues stress the variability of the sulcal patterning in the temporal lobe that they then referred to as a "bewildering diversity" in their atlas of the human brain a year later (Bonin et al. 1951^68^; see “Discussion”).
4. *The MFS was mentioned, but not in the context of the human or chimpanzee brain.* For example, while Connolly (1950) examined the sulcal patterning in 28 chimpanzee hemispheres (across 17 chimpanzee brains), he mentioned "Retzius' sulcus of the fusiform gyrus" in the context of the baboon brain, but not in reference to the chimpanzee or human brain. Connolly writes:
  "*When the caudal end of the occipito-temporal turns laterally or a larger fusiform gyrus forms by the extent and direction of the collateral *(*figure 47, left hemisphere*),* a sagittal sulcus is formed, often proceeding from the transverse occipital. This is Retzius' sulcus of the fusiform gyrus. A further variation is seen when this sulcus along with the collateral and the occipito-temporal runs approximately parallel, but this is more frequently seen in the large anthropoids*." (*P. 61*).
5. *The MFS is not mentioned and while the fusiform is discussed briefly, it is obscured by the cerebellum in photographs*. Walker and Fulton (1936) examined 29 hemispheres of the chimpanzee brain and mention the fusiform once in the text. They write:
  *"The lobulus fusiformis lies between the inferior temporal and the collateral fissures, and the lobulus lingualis between the collateral and hippocampal fissures. The divisions between these are however frequently indefinite." P. 111.*

Furthermore, in 10 figures across 5 plates, the FG is labeled in one hemisphere, but the sulcal patterning of the FG is blocked by the cerebellum. In the one plate in which the FG is exposed, while the view is medial, the sulcal patterning resembles the patterning identified in our present sample (Fig. 9). Nevertheless, from this view, what we believe to be the MFS cannot definitively be differentiated from the OTS.

Altogether, the findings from the present study reveal that the sulcal definitions in non-human hominoids are more consistent than reported by classic studies, which require a reconsideration of the sulcal patterning within ventral occipito-temporal cortex of the non-human hominoid brain that incorporates the MFS.
