# Supplementary material for: Sulcal morphology of ventral temporal cortex is shared between humans and other hominoids
Source: Sci Rep. 2020 Oct 13;10:17132. doi: 10.1038/s41598-020-73213-x (PMC7555511; doi:10.1038/s41598-020-73213-x)
Supplement: Supplementary file 1 — Supplementary Figures. [file 41598_2020_73213_MOESM1_ESM.pdf]

Supplementary Information

Sulcal morphology of ventral temporal cortex is shared between humans and other hominoids

Jacob A. Miller[1], Willa I. Voorhies[2], Xiang Li[3], Ishana Raghuram[2], Nicola Palomero-Gallagher[4,5,6], Karl Zilles[4,7], Chet C. Sherwood[8], William D. Hopkins[9], Kevin S. Weiner[1,2]

[1] Helen Wills Neuroscience Institute, University of California, Berkeley, Berkeley CA, 94720 USA
[2] Department of Psychology, University of California, Berkeley, Berkeley CA, 94720 USA
[3] School of Clinical Sciences, University of Edinburgh, Edinburgh, UK
[4] Research Centre Jülich, Institute of Neuroscience and Medicine INM-1, Jülich, Germany
[5] Department of Psychiatry, Psychotherapy and Psychosomatics, Medical Faculty, RWTH Aachen University, Aachen
[6] C. & O. Vogt Institute for Brain Research, Heinrich-Heine-University, 40225 Düsseldorf, Germany
[7]JARA-Translational Brain Medicine, Aachen, Germany
[8] Department of Anthropology and Center for the Advanced Study of Human Paleobiology, The George Washington University, 800 22nd Street NW, Suite 6000, Washington, DC, 20052, USA.
[9] Department of Comparative Medicine, The University of Texas MD Anderson Cancer Center, Bastrop, TX, 78602

Corresponding Author:

Jacob A. Miller, Helen Wills Neuroscience Institute, 210 Barker Hall, UC Berkeley, Berkeley CA, 94720, jacob_miller@berkeley.edu

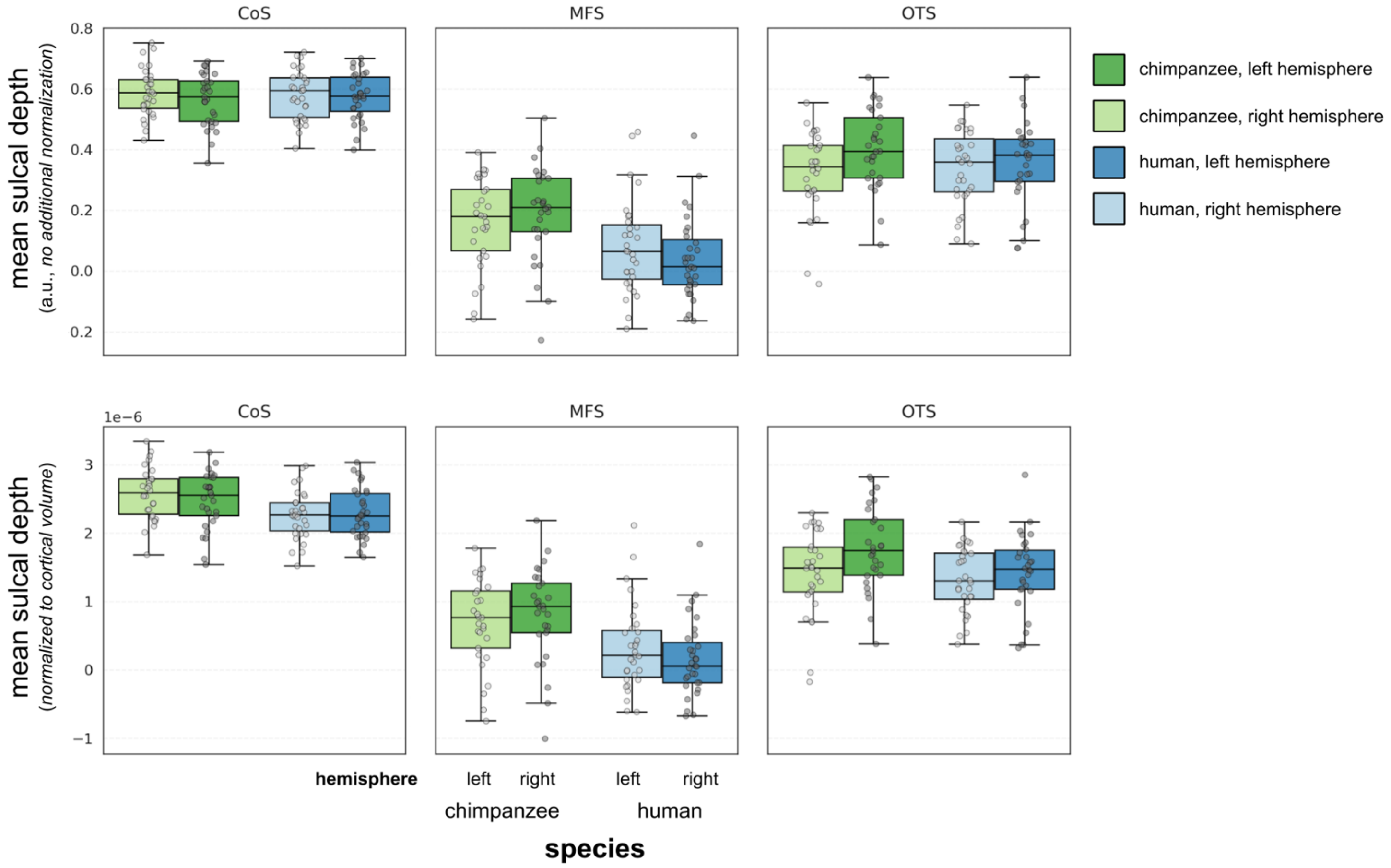

Supplementary Figure 1. The MFS is relatively deeper in chimpanzees compared to humans, regardless of normalization metric or method. (A) Box plots indicating the mean sulcal depth (from FreeSurfer) for the collateral sulcus (CoS; left), mid-fusiform sulcus (MFS; middle), and the occipito-temporal sulcus (OTS; right) for the left (darker shade) and right (lighter shade) hemispheres in humans (blue) and chimpanzees (green). **(B)** Same layout, but for mean sulcal depth normalized by the total cortical gray matter volume within each hemisphere. For both metrics, the MFS is relatively deeper in chimpanzees than in humans.

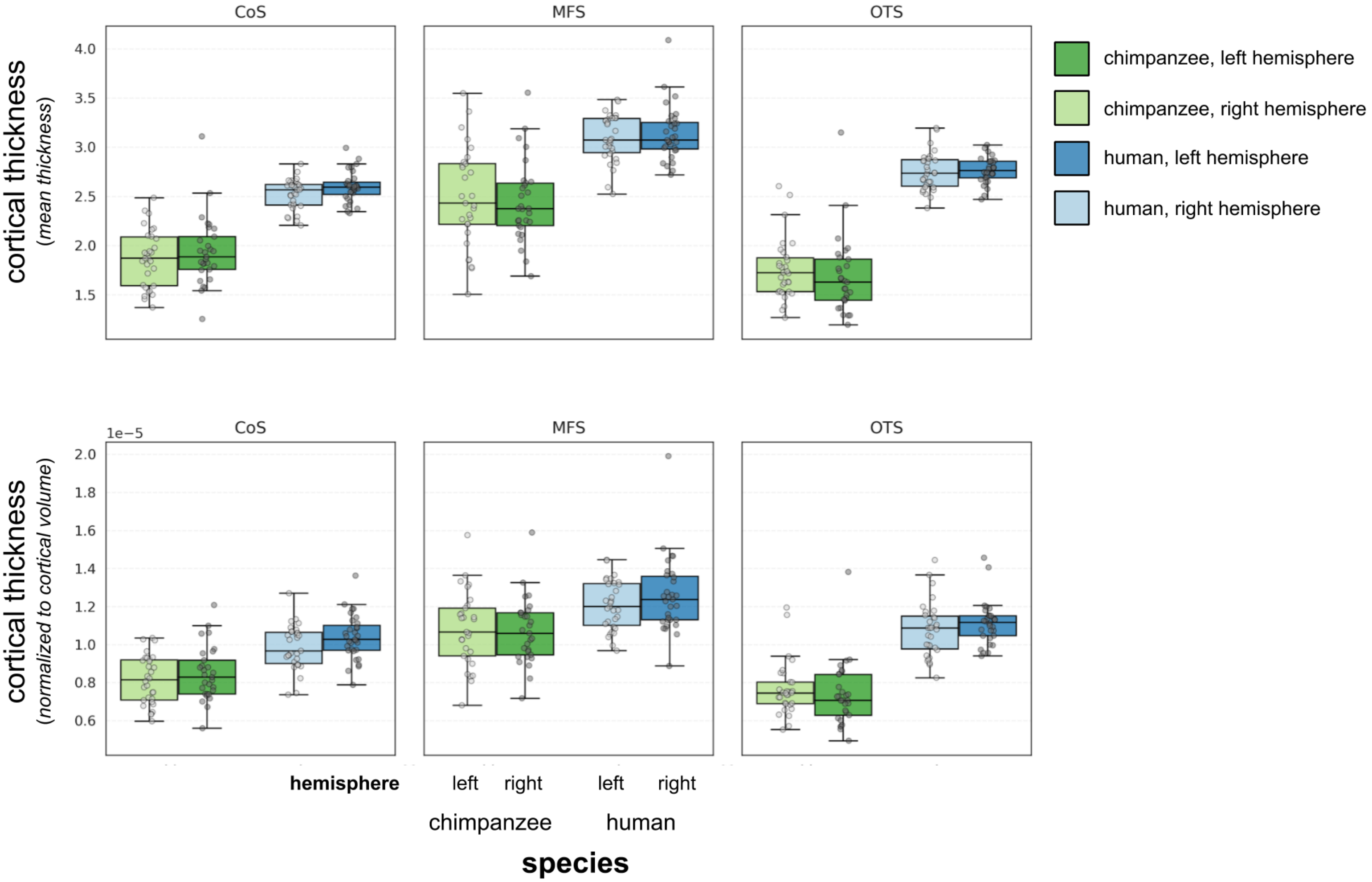

Supplementary Figure 2. Sulci in ventral temporal cortex are thinner in chimpanzees compared to humans, regardless of normalization method or metric. (**A**) Box plots indicating the mean cortical thickness (from FreeSurfer, no normalization) for CoS (left), MFS (middle), and OTS (right) for the left (darker shade) and right hemispheres (light shade) in humans (blue) and chimpanzees (green). The CoS, MFS, and OTS are all thinner in chimpanzees compared to humans. **(B)** Same layout, but for mean cortical thickness normalized by the total cortical gray matter volume within each hemisphere. For both metrics, all sulci are relatively thicker in the human versus chimpanzee cortex.

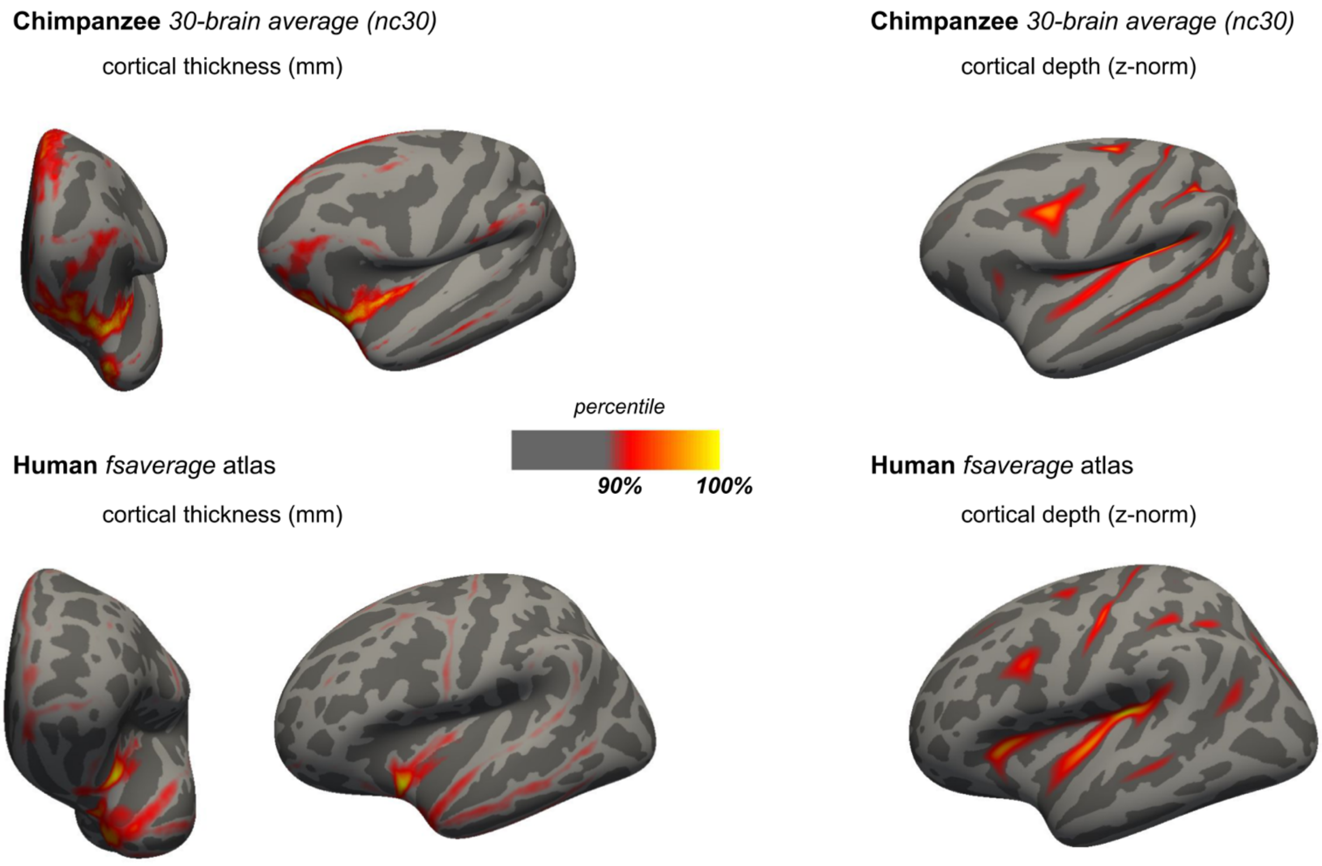

Supplementary Figure 3. Cortical thickness and depth in chimpanzee and human cortical templates. *Left*, points of maximum (above 90th percentile) cortical thickness (mm) across the cortex show consistency across species (*top*, chimpanzee; *bottom*, human), with the insular cortex being the thickest area. *Right*, same as *Left*, but for points of maximum sulcal depth across cortex (normalized units) in chimpanzee and human template brains. Chimpanzee template was generated in a held-out sample of 30 anatomical scans (Methods).

Hylobates lar

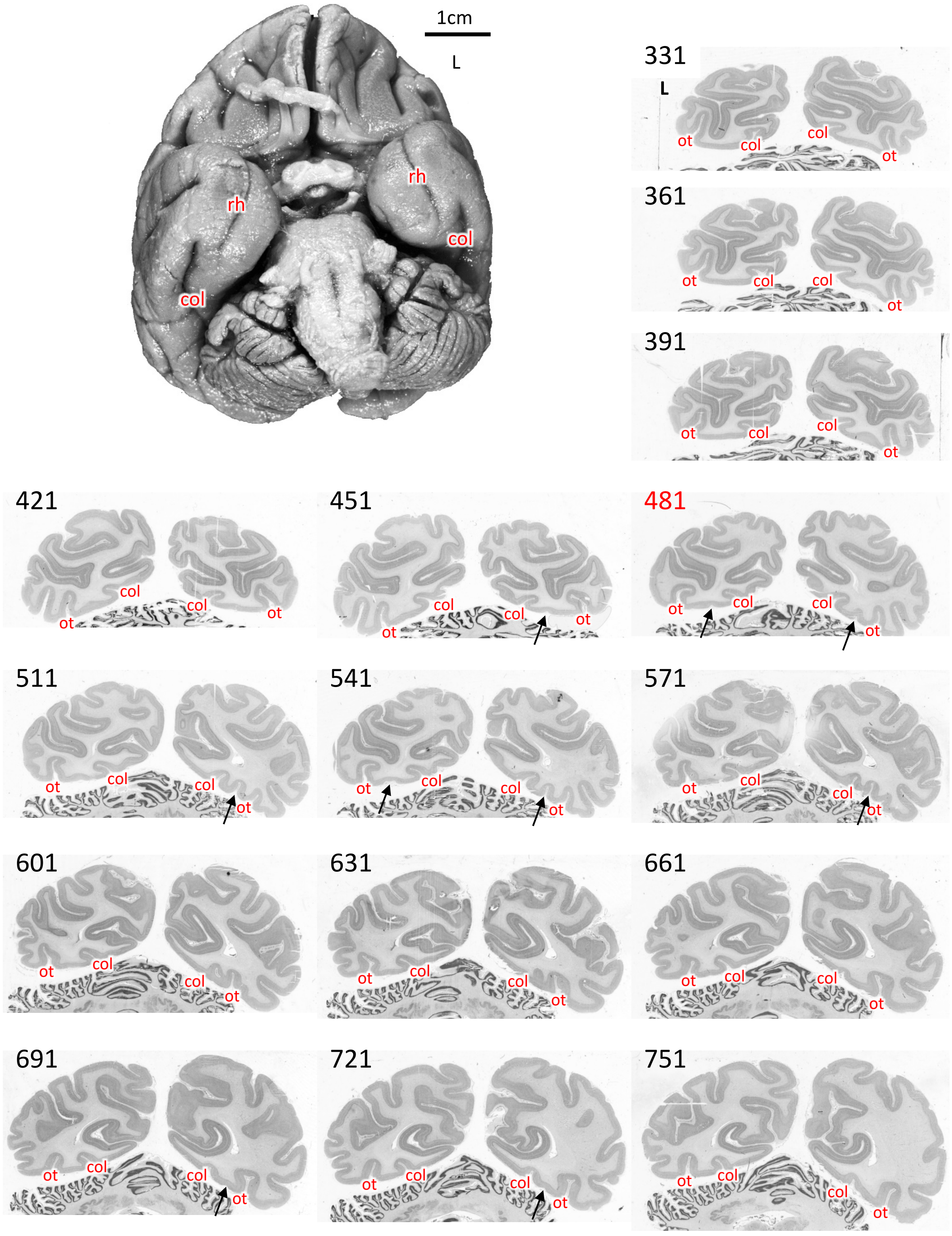

Supplementary Figure 4. **The mid-fusiform sulcus in the gibbon *(Hylobates lar).*** The shallowness of the MFS relative to surrounding sulci (CoS, OTS) generates a distinctive pattern on histological coronal sections. We leveraged this fact to identify the MFS in not only humans and chimpanzees as in the prior analyses, but also additional hominoids (bonobos, gorillas, orangutans, and gibbons). This Figure is an extension of Figure 6. The top left image is a photograph of the brain from a ventral view. The rhinal (rh) and collateral (col) sulci are labeled. Several single histological sections are then included with collateral (col), mid-fusiform (MFS, arrow), and occipito-temporal (ot) sulci labeled. Serial coronal sections through the occipital lobe of *Hylobates lar* (brain ID YN81-146). Section highlighted in red is shown in Fig. 6. Distance between sections can be determined by multiplying the difference between two section numbers by the section thickness (20µm). col: collateral sulcus; L: left; ot: occipito-temporal sulcus; rh: rhinal sulcus. Scale bar applies to the brain photograph and to histological sections.

Pan troglodytes

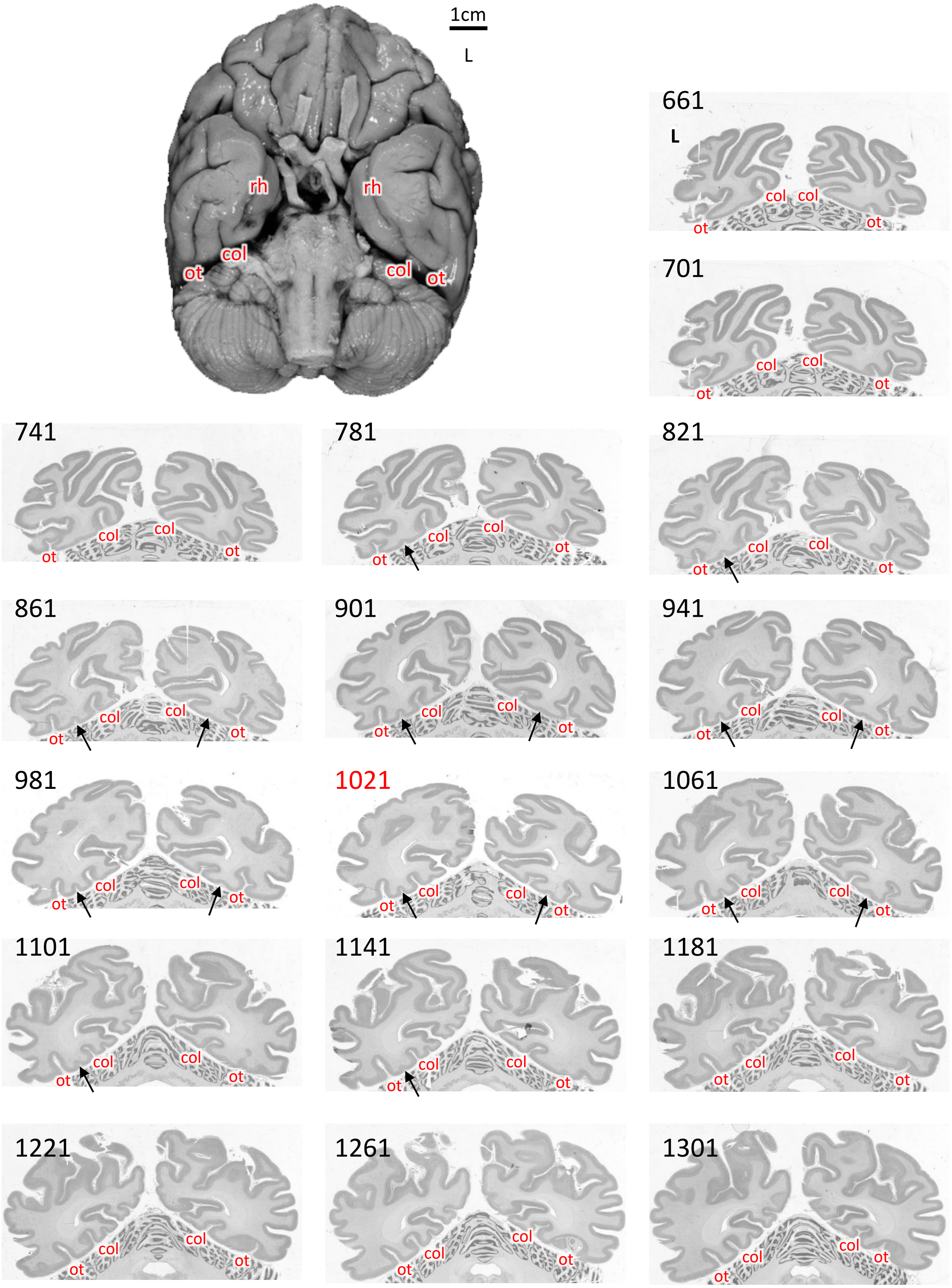

Supplementary Figure 5. The mid-fusiform sulcus in the chimpanzee *(Pan troglodytes).* Same layout as Figure S1. Section highlighted in red is shown in Fig. 6. Distance between sections can be determined by multiplying the difference between two section numbers by the section thickness (20µm). col: collateral sulcus; L: left; ot: occipito-temporal sulcus; rh: rhinal sulcus. Scale bar applies to the brain photograph and to histological sections. Serial coronal sections through the occipital lobe of *Pan troglodytes* (brain ID 4/97). Arrow: mid-fusiform sulcus.

Pan paniscus

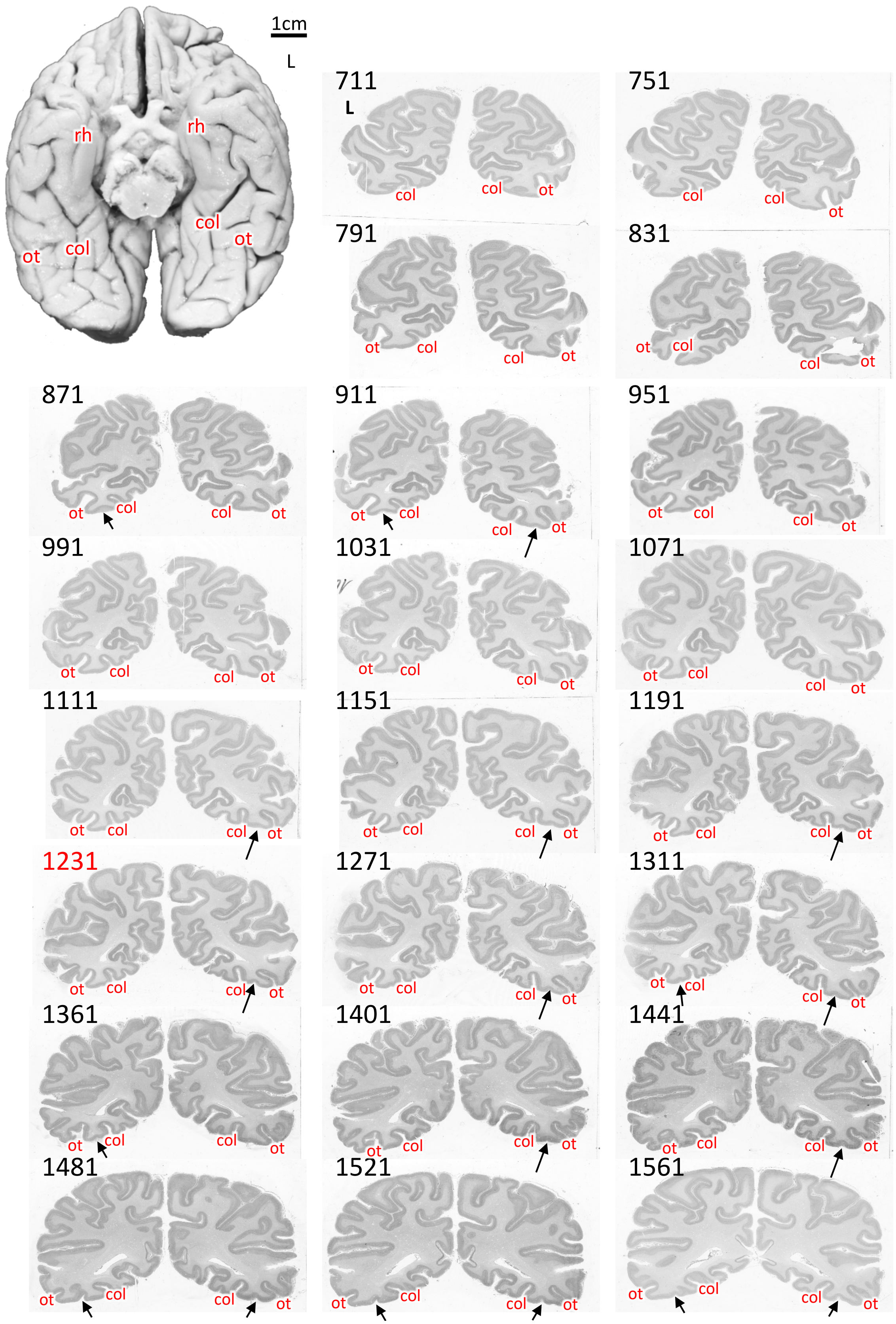

Supplementary Figure 6. The mid-fusiform sulcus in the bonobo *(Pan paniscus)*. Same layout as Figure S1. Section highlighted in red is shown in Fig. 6. Distance between sections can be determined by multiplying the difference between two section numbers by the section thickness (20µm). col: collateral sulcus; L: left; ot: occipito-temporal sulcus; rh: rhinal sulcus. Scale bar applies to the brain photograph and to histological sections. Serial coronal sections through the occipital lobe of *Pan paniscus* (brain ID Pan1). Arrow: mid-fusiform sulcus.

Gorilla gorilla

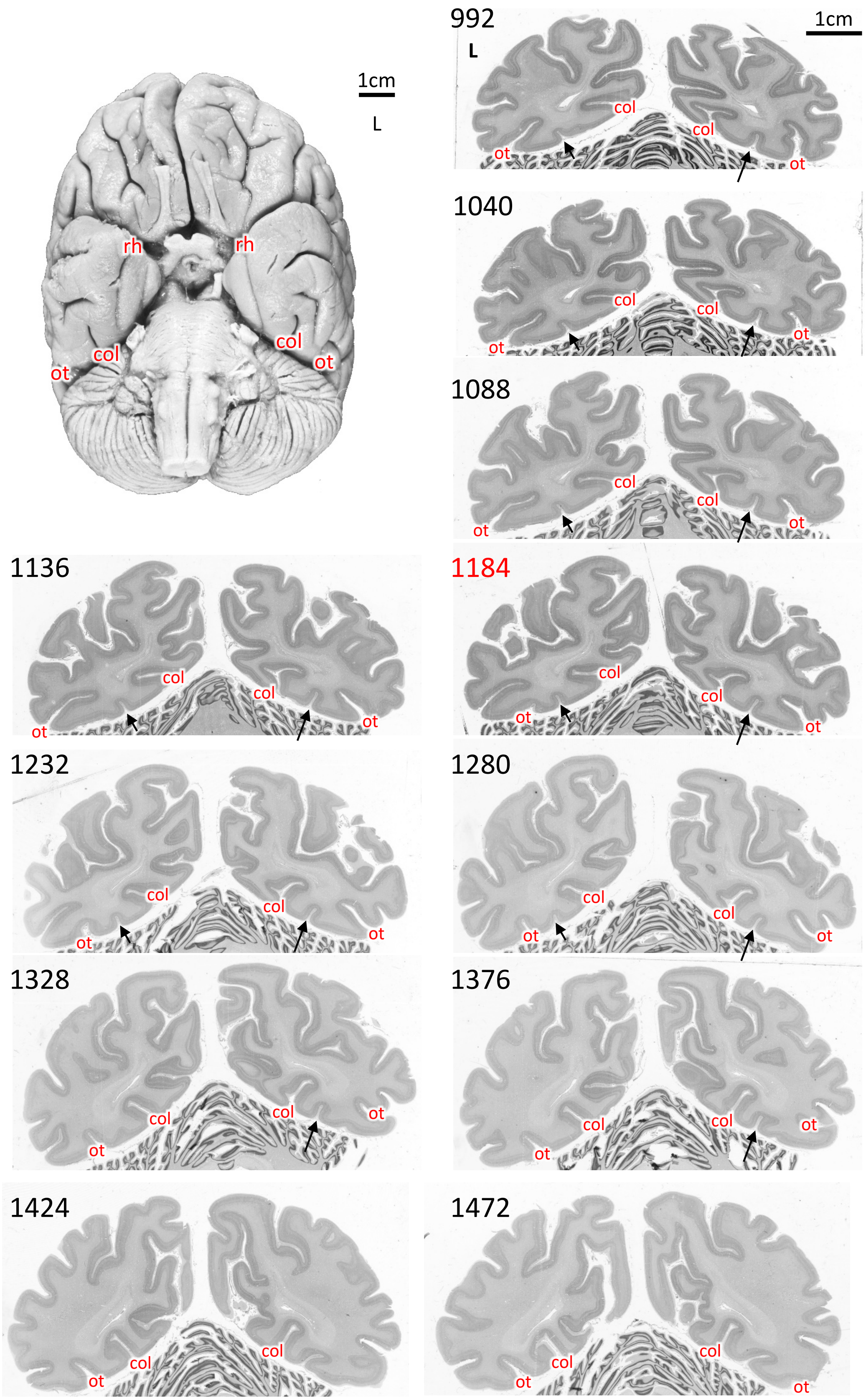

Supplementary Figure 7. The mid-fusiform sulcus in the gorilla *(Gorilla gorilla).* Same layout as Figure S1. Section highlighted in red is shown in Fig. 6. Distance between sections can be determined by multiplying the difference between two section numbers by the section thickness (20µm). col: collateral sulcus; L: left; ot: occipito-temporal sulcus; rh: rhinal sulcus. Scale bar applies to the brain photograph and to histological sections. Serial coronal sections through the occipital lobe of *Gorilla gorilla* (brain ID YN82-140). Arrow: mid-fusiform sulcus.

Pongo pygmaeus

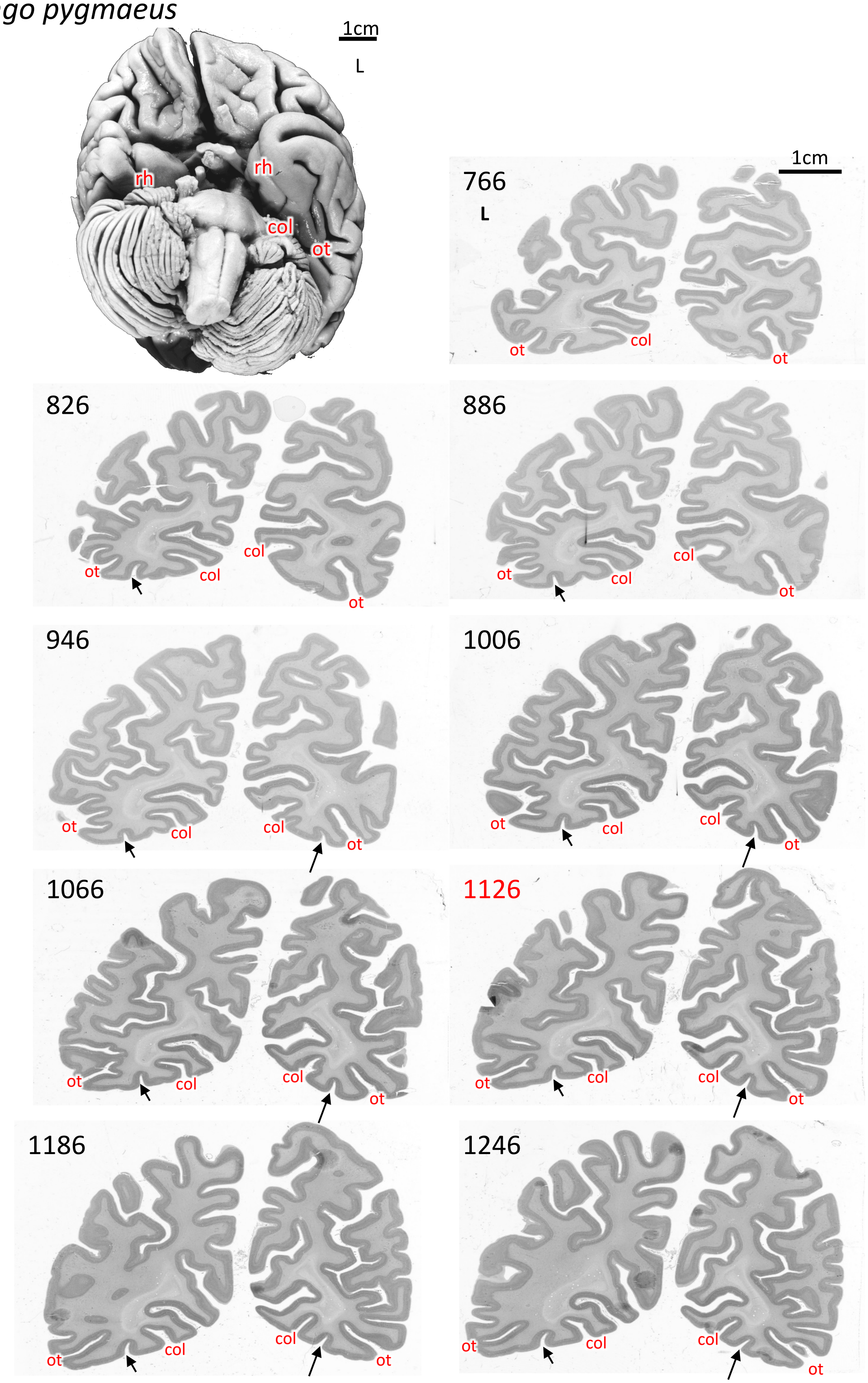

Supplementary Figure 8. The mid-fusiform sulcus in the orangutan *(Pongo pygmaeus)*. Same layout as Figure S1. Section highlighted in red is shown in Fig. 6. Distance between sections can be determined by multiplying the difference between two section numbers by the section thickness (20µm). col: collateral sulcus; L: left; ot: occipito-temporal sulcus; rh: rhinal sulcus. Scale bar applies to the brain photograph and to histological sections. Serial coronal sections through the occipital lobe of *Pongo pygmaeus* (brain ID YN 85-38). Arrow: mid-fusiform sulcus.
